# Defective immuno- and thymoproteasome assembly causes severe immunodeficiency

**DOI:** 10.1038/s41598-018-24199-0

**Published:** 2018-04-13

**Authors:** Irina Treise, Eva M. Huber, Tanja Klein-Rodewald, Wolfgang Heinemeyer, Simon A. Grassmann, Michael Basler, Thure Adler, Birgit Rathkolb, Laura Helming, Christian Andres, Matthias Klaften, Christina Landbrecht, Thomas Wieland, Tim M. Strom, Kathy D. McCoy, Andrew J. Macpherson, Eckhard Wolf, Marcus Groettrup, Markus Ollert, Frauke Neff, Valerie Gailus-Durner, Helmut Fuchs, Martin Hrabě de Angelis, Michael Groll, Dirk H. Busch

**Affiliations:** 1 Institute for Medical Microbiology, Immunology and Hygiene, Technical University of Munich, Trogerstr. 30, 81675 Munich, Germany; 2 Center for Integrated Protein Science at the Department Chemistry, Chair of Biochemistry, Technical University of Munich, Lichtenbergstr. 4, 85748 Garching, Germany; 3 German Mouse Clinic, Institute of Experimental Genetics, Helmholtz Zentrum München, Ingolstädter Landstr. 1, 85764 Neuherberg, Germany; 4 Institute for Molecular Animal Breeding and Biotechnology, Gene Center of the Ludwig-Maximilians-Universität München, Feodor-Lynen Str. 25, 81377 Munich, Germany; 5 University Clinic of Visceral Surgery and Medicine, Departement Klinische Forschung, University of Bern, Murtenstr. 35, CH-3010 Bern, Switzerland; 6 Institute of Human Genetics, Helmholtz Zentrum München, Ingolstädter Landstr. 1, 85764 Neuherberg, Germany; 7 Institute of Pathology, Helmholtz Zentrum München, Ingolstädter Landstr. 1, 85764 Neuherberg, Germany; 8 Division of Immunology, Department of Biology, University of Konstanz, Universitaetsstr. 10, 78457 Konstanz, Germany; 9 Department of Dermatology and Allergy Biederstein, Technical University of Munich, Biedersteiner Str. 29, 80802 Munich, Germany; 10 Focus Group “Clinical Cell Processing and Purification”, Institute for Advanced Study, Technical University of Munich, 81675 Munich, Germany; 11 German Center for Infection Research (DZIF), Trogerstr. 30, 81675 Munich, Germany; 12 Chair of Experimental Genetics, Center of Life and Food Sciences Weihenstephan, Technical University of Munich, 85354 Freising, Weihenstephan Germany; 13 German Center for Diabetes Research (DZD), Ingostädter Landstr. 1, 85764 Neuherberg, Germany; 14 Department of Infection and Immunity, Luxembourg Institute of Health, 29 rue Henri Koch, L-4354 Esch-sur-Alzette, Luxembourg; 15 Department of Dermatology and Allergy Center, Odense Research Center for Anaphylaxis, University of Southern Denmark, DK-5000 Odense C, Denmark; 16 Institute of Human Genetics, Technical University of Munich, 81675 Munich, Germany

## Abstract

By N-ethyl-N-nitrosourea (ENU) mutagenesis, we generated the mutant mouse line TUB6 that is characterised by severe combined immunodeficiency (SCID) and systemic sterile autoinflammation in homozygotes, and a selective T cell defect in heterozygotes. The causative missense point mutation results in the single amino acid exchange G170W in multicatalytic endopeptidase complex subunit-1 (MECL-1), the β2i-subunit of the immuno- and thymoproteasome. Yeast mutagenesis and crystallographic data suggest that the severe TUB6-phenotype compared to the MECL-1 knockout mouse is caused by structural changes in the C-terminal appendage of β2i that prevent the biogenesis of immuno- and thymoproteasomes. Proteasomes are essential for cell survival, and defective proteasome assembly causes selective death of cells expressing the mutant MECL-1, leading to the severe immunological phenotype. In contrast to the immunosubunits β1i (LMP2) and β5i (LMP7), mutations in the gene encoding MECL-1 have not yet been assigned to human disorders. The TUB6 mutant mouse line exemplifies the involvement of MECL-1 in immunopathogenesis and provides the first mouse model for primary immuno- and thymoproteasome-associated immunodeficiency that may also be relevant in humans.

## Introduction

The 26S proteasome is the major non-lysosomal protease engaged in degradation of intracellular proteins. Beyond basic protein homeostasis, proteasomes have wide-spread functions such as control of signalling pathways via selective proteolysis^1^ and generation of peptides for major histocompatibility complex (MHC) class-I presentation^2^. The heart of each 26S proteasome is the 20S core particle (CP), consisting of seven different α- and seven different β-subunits, arranged in a cylindrical complex of four stacked rings following an α_1–7_β_1–7_β_1–7_α_1–7_ stoichiometry. The proteolytically active sites reside in the β1-, β2-, and β5-subunits and are buried on the inner side of the protease. The principal proteasome structure and its assembly pathway are highly conserved among species from yeast to mammals^3,4^. However, contrary to yeast, which harbours only one proteasome (yCP), mammals express three different types of CPs with distinct enzymatically active β-subunit compositions: the constitutive proteasome (cCP: β1c, β2c and β5c), the immunoproteasome (iCP: β1i (also known as LMP2), β2i (also known as MECL-1) and β5i (also known as LMP7)) and the thymoproteasome (tCP: β1i, β2i and β5t)^5^. The iCP is predominantly produced in lymphocytes, but its expression can be induced also in non-immune tissues by the pro-inflammatory cytokine interferon (IFN)-γ^6,7^. The iCP generates the majority of MHC class-I antigens required for efficient immune surveillance and pathogen clearance by CD8^+^ T cells as proven by knockout (KO) mice lacking individual^8–10^ or all three catalytic immunosubunits^11^. Biogenesis of the tCP is restricted to cortical thymic epithelial cells (cTECs) and β5t KO studies demonstrated that this type of CP is vital for positive selection during the CD8^+^ T cell maturation process^12,13^. Besides the cCP, iCP and tCP, mixed-type CPs incorporating the subunits β1i, β2c and β5i or β1c, β2c and β5i were identified^14–16^.

Due to the essential functions of the proteasome, mutations in any of its components are associated with diverse human diseases such as diabetes, cancer and autoinflammation^17,18^. Most proteasome-associated autoinflammatory syndromes (PRAAS), also known as autoinflammation, lipodystrophy and dermatosis (ALDD, OMIM entry 256040), are linked to mutations in the *PSMB8* gene, encoding the iCP subunit β5i^19–22^. However, amino acid exchanges in other proteasome subunits such as α7, β7 and β1i were identified as well^23^. Although the pathogenic mechanisms are not yet completely resolved, CP assembly and activity defects are found in these patients irrespective of the underlying mutations^22,23^.

Biogenesis of the eukaryotic CP is a well-organised and highly controlled process, involving several chaperones^24,25^. It starts with the formation of the α-ring from the subunits α1- α7. The β-subunits subsequently use the heteroheptameric α-ring as a docking platform to assemble in a well-defined order resulting in an α_1–7_β_1–7_ precursor complex (half-CP). Dimerisation of two half-CPs finally triggers autocatalytic removal of the propeptides from the β-subunits to yield a mature proteasome^26,27^. One of the first β-subunits that associate with the α_1–7_ ring is β2^15,28^. Its long C-terminal tail is essential for viability in yeast and human cells by recruiting and positioning the adjacent β3 subunit^29^. Therefore, the β2 appendage is highly conserved in structure and sequence among eukaryotic species as well as constitutive and immuno-β2-subunits^4,26^. Once the CP is assembled, its subunit composition is unchangeable; hence, intracellular levels of CP types have to be regulated by *de novo* proteasome synthesis^30^. Nevertheless, how the incorporation of constitutive vs. alternative subunits is controlled is still unclear.

In a large-scale mouse N-ethyl-N-nitrosourea (ENU) mutagenesis and phenotypic screening approach, aimed at discovering mutant mouse lines with clinical phenotypes as models for human diseases^31^, we identified the single amino acid substitution G170W in the proteasome subunit MECL-1 to cause severe combined immunodeficiency (SCID) and systemic autoinflammation. Yeast mutagenesis as well as X-ray crystallographic data suggest that expression of the mutant β2i subunit aborts the biogenesis of iCPs and tCPs and is lethal to immune cells, which ultimately manifests in lymphopenia. Altogether, the here described mutation links for the first time an autoinflammatory syndrome to the β2i subunit of iCP and tCP and serves as a first mouse model for human ALDD/PRAAS diseases.

## Results

### Heterozygous TUB6 mutants are characterised by a predominant T cell defect

**Figure 1:**
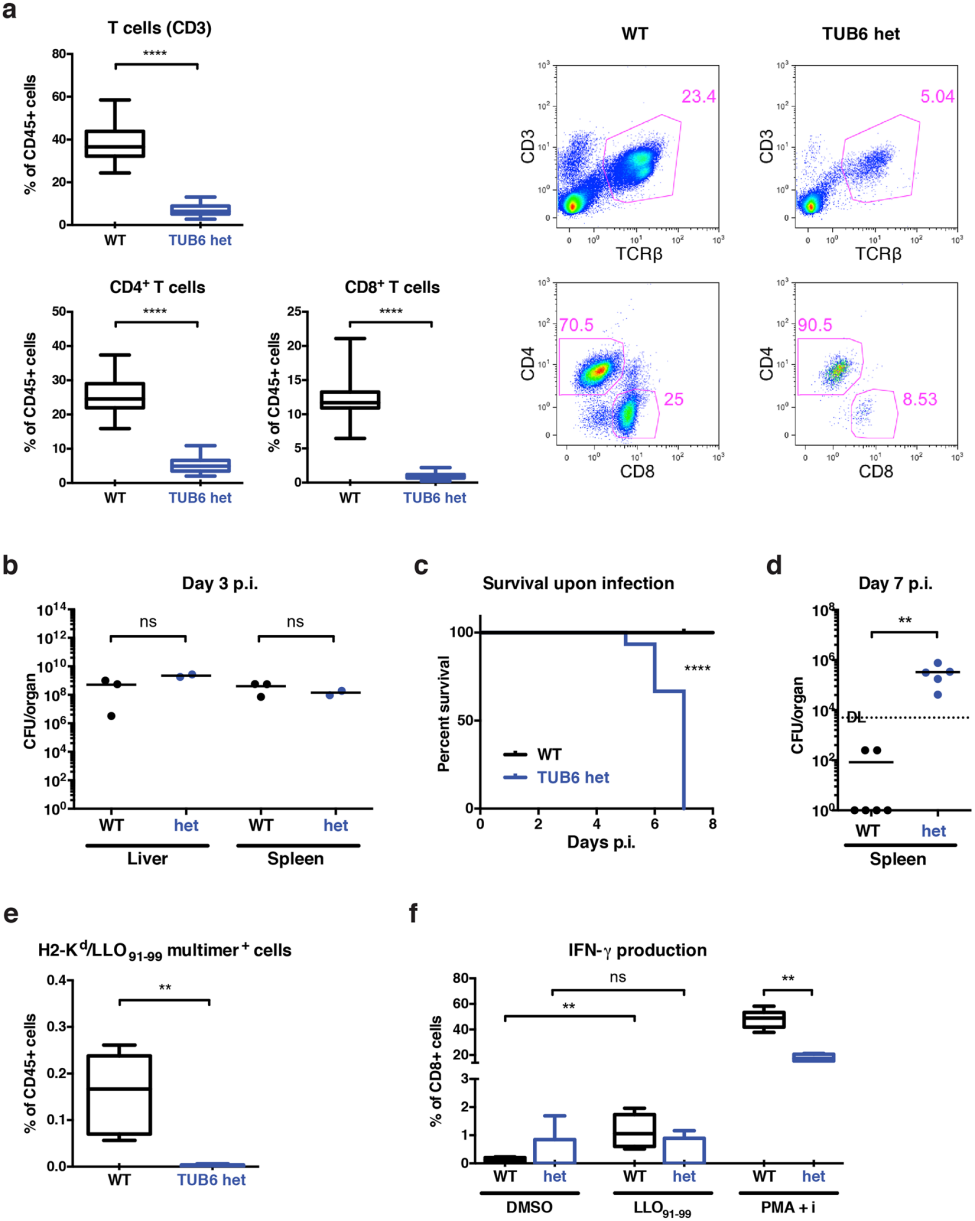
Phenotype of heterozygous TUB6 mutants characterised by numerical and functional T cell defect. (**a**) T cell frequencies in peripheral blood from heterozygous mutants and littermate controls shown as box plots (pooled data from different experiments, WT n = 40, het n = 71) and representative dot plots (upper panel, gated on living CD45^+^ cells), and CD4 versus CD8 profiles of T cells (lower panel, gated on living CD45^+^ CD3^+^ TCRβ^+^ cells). (**b**–**f**) Challenge of heterozygous TUB6 mutants with a low dose of 5000 colony forming units (CFU) (**b**) or 1000 CFU (**c**–**f**) *Listeria*. (**b**,**d**) Bacterial loads in indicated organs from infected mice on day 3 (**b**) and day 7 (**d**) p.i. Points indicate individual mice. DL detection limit. (**c**) Survival of infected mice. ****P < 0.0001, Log-rank (Mantel-Cox) test. (**e**,**f**) Analysis of antigen-specific T cell response on day 7 p.i. Relative proportions of H-2K^d^/LLO_91–99_ multimer^+^ cells as percentages of total splenocytes (**e**) and IFN-γ expressing CD8^+^ T cells in response to stimulation with dimethyl sulfoxide (DMSO, negative control), peptide LLO_91–99_ or phorbol 12-myristate 13-acetate (PMA) and ionomycin (**f**). For flow cytometry dot plots please refer to Supplementary Fig. S1.

For discovering novel genes or gene functions, mouse mutagenesis models have proven to be invaluable^32^. The powerful mutagen ENU induces genome-wide point mutations^33^, mirroring human single nucleotide variants causative for numerous monogenic disorders. As part of the ENU mutagenesis program, we analysed within the Immunology module of the German Mouse Clinic peripheral blood of F1 offspring from ENU-treated mice to identify mutants with immunological defects^31,34–36^. The founder animal of mutant mouse line TUB6 was selected by the phenotype of significantly decreased proportions of CD4^+^ and CD8^+^ T cells in peripheral blood and an increase of the CD4/CD8 ratio (Fig. 1a). Further breeding revealed this phenotype to fully penetrate in heterozygous TUB6 mutants. This allowed for a clear differentiation from wild type littermates; otherwise heterozygotes did not display any visually apparent physical abnormalities. Body weights and immunoglobulin subtypes IgM, IgA, IgG1, IgG2a, IgG2b, IgG3 were within the range of wild type littermates (Supplementary Fig. S1). B-, NK cells and granulocytes were slightly increased in relative proportions, but not in absolute numbers, showing that the increase is relative to the decrease of T cells (Supplementary Fig. S1).

To assess the immune competence of heterozygous TUB6 mutants, we evaluated the immune response to a sublethal dose of *Listeria monocytogenes* (*L*.*m*.). In the early phase of infection (day 3), bacterial burden in spleens and livers of heterozygous mutants did not differ significantly from infected controls (Fig. 1b). On days 5–7 post infection (p.i.), however, all heterozygous mice failed to control the infection, became moribund and had to be euthanised due to ethical reasons, while all littermate controls showed good health conditions (Fig. 1c). On day 7 p.i., analysis of the euthanised heterozygotes showed failure to eliminate the pathogen. Considerable numbers of living bacteria were recoverable from mutant spleen tissue, whereas wild type mice showed efficient clearance (Fig. 1d). Since lack of *L*.*m*. clearance points towards a T cell defect, we investigated pathogen-specific T cell responses in these mice. In heterozygous TUB6 splenocytes, we could hardly detect any H-2K^d^/LLO_91–99_^+^ CD8^+^ T cells (Fig. 1e). Congruently, CD8^+^ T cells from heterozygous TUB6 mice failed to produce IFN-γ in response to restimulation with LLO_91–99_ peptide, whereas a significant IFN-γ^+^ induction in CD8^+^ T cells from wild type mice was observed (Fig. 1f). CD8^+^ T cells from heterozygous TUB6 mice, however, were not generally deficient in cytokine production, as demonstrated by the ability of T cells to produce IFN-γ in response to antigen-independent stimulation with phorbol 12-myristate 13-acetate (PMA) and ionomycin (Fig. 1f).

In summary, in heterozygous TUB6 mice a selective T cell defect was observed, with a substantially reduced T cell compartment at baseline conditions and failure of developing a protective antigen-specific T cell response to *L*.*m*. infection.

### Homozygous mutant TUB6 mice have a reduced life span and severe combined immunodeficiency (SCID)

**Figure 2:**
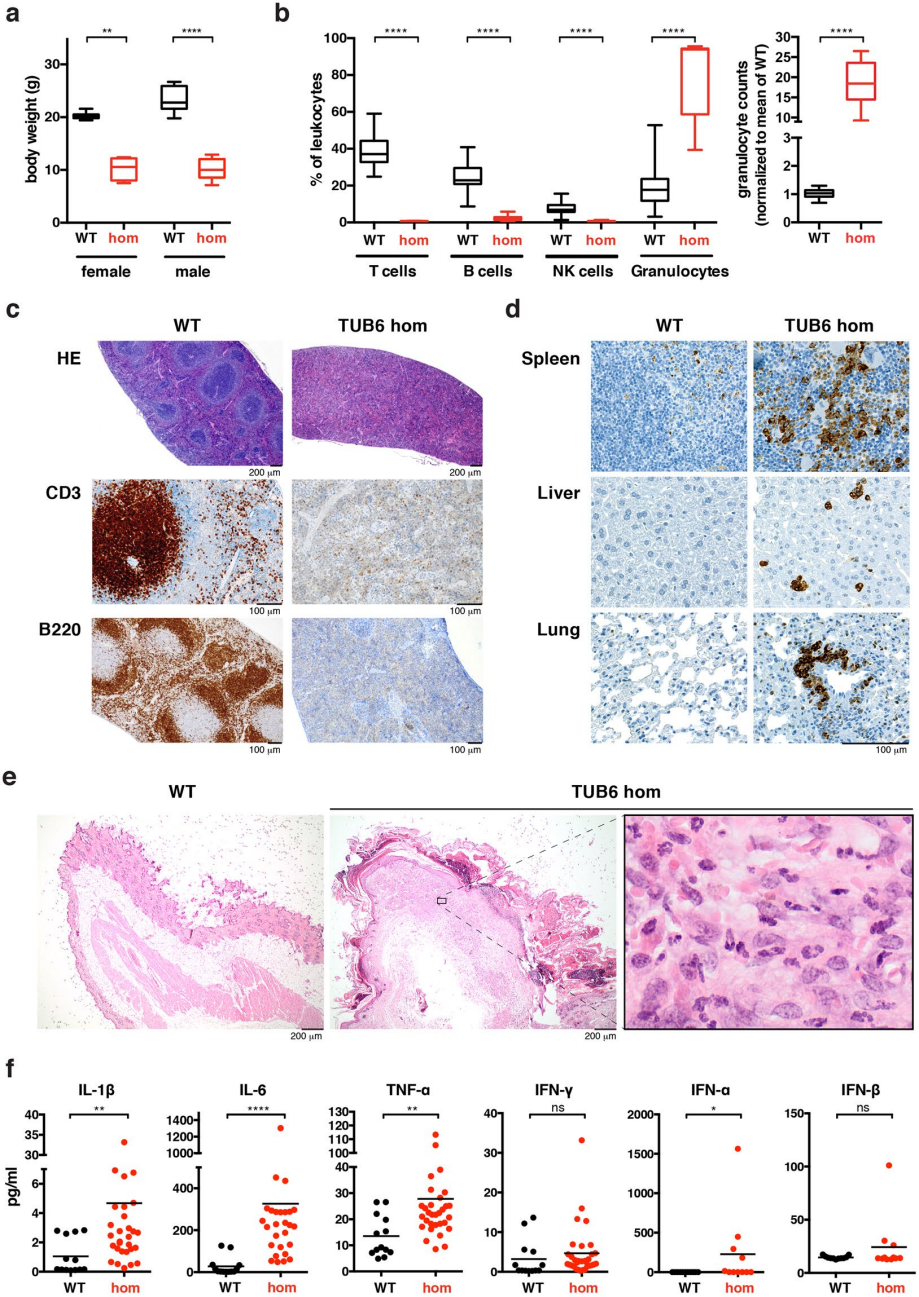
Phenotypic comparison of 8-week-old homozygous TUB6 mutants and wild type littermates. (**a**) Body weight data (n = 8–10 per group). (**b**) Flow cytometric profile of peripheral blood leukocytes (pooled, hom n = 11, WT n = 44). The right box plot shows granulocyte counts after equal acquisition time per sample, normalised to the mean of wild types. For flow cytometry dot plots see Supplementary Fig. S2. (**c**–**e**) Representative (immuno-)histological images of spleen (**c**), neck skin **(e**) and further indicated organs (**d**) stained with HE (**c**,**e**), anti-CD3 or anti-B220 (**c**) as indicated, or anti-MPO (**d**). (**f**) Cytokine concentration in plasma.

Upon heterozygous intercross, about 25% of the offspring revealed a significantly smaller body size (Fig. 2a) and a moribund appearance by 6 to 12 weeks of age. Flow cytometric analysis of peripheral blood demonstrated that homozygous TUB6 mice were profoundly lymphopenic and displayed T^−^ B^−^ NK^−^ SCID (Fig. 2b). Residual T cells were predominantly CD4^+^; CD8^+^ T cells comprised only 0.002% of all splenocytes (Supplementary Fig. S2). As predicted for lymphocyte deficiency, thymus and lymph nodes were not detectable, while spleens were devoid of lymphocytes and lacked the normal red and white pulp morphology (Fig. 2c). Consistent with the B cell deficiency, immunoglobulin levels were below the detection limit (data not shown).

Proportions as well as total numbers of granulocytes were immensely increased in peripheral blood from homozygous mutants (Fig. 2b). Microscopy of blood smears confirmed that most leukocytes represented neutrophil granulocytes (Supplementary Fig. S2). Granulocyte infiltrates in varying degrees were detected in liver, spleen and lungs, as shown by myeloperoxidase (MPO) staining of tissue sections (Fig. 2d). Furthermore, homozygous mutants developed a progressive skin disorder characterised by hyperkeratosis, alopecia, lack of adipose tissue, and infiltration of neutrophil granulocytes (Fig. 2e). The pro-inflammatory cytokines IL-1β, IL-6 and TNF-α were strongly increased in plasma from homozygous mutants, while IFN-γ, -α and -β were increased only in some individual mice (Fig. 2f).

Taken together, homozygous TUB6 mice exhibit a lethal immunological phenotype comprising SCID and systemic inflammation.

### T and NK cell deficiency is inborn, while B cell deficiency and granulocytosis develop later in a commensal-independent manner

**Figure 3:**
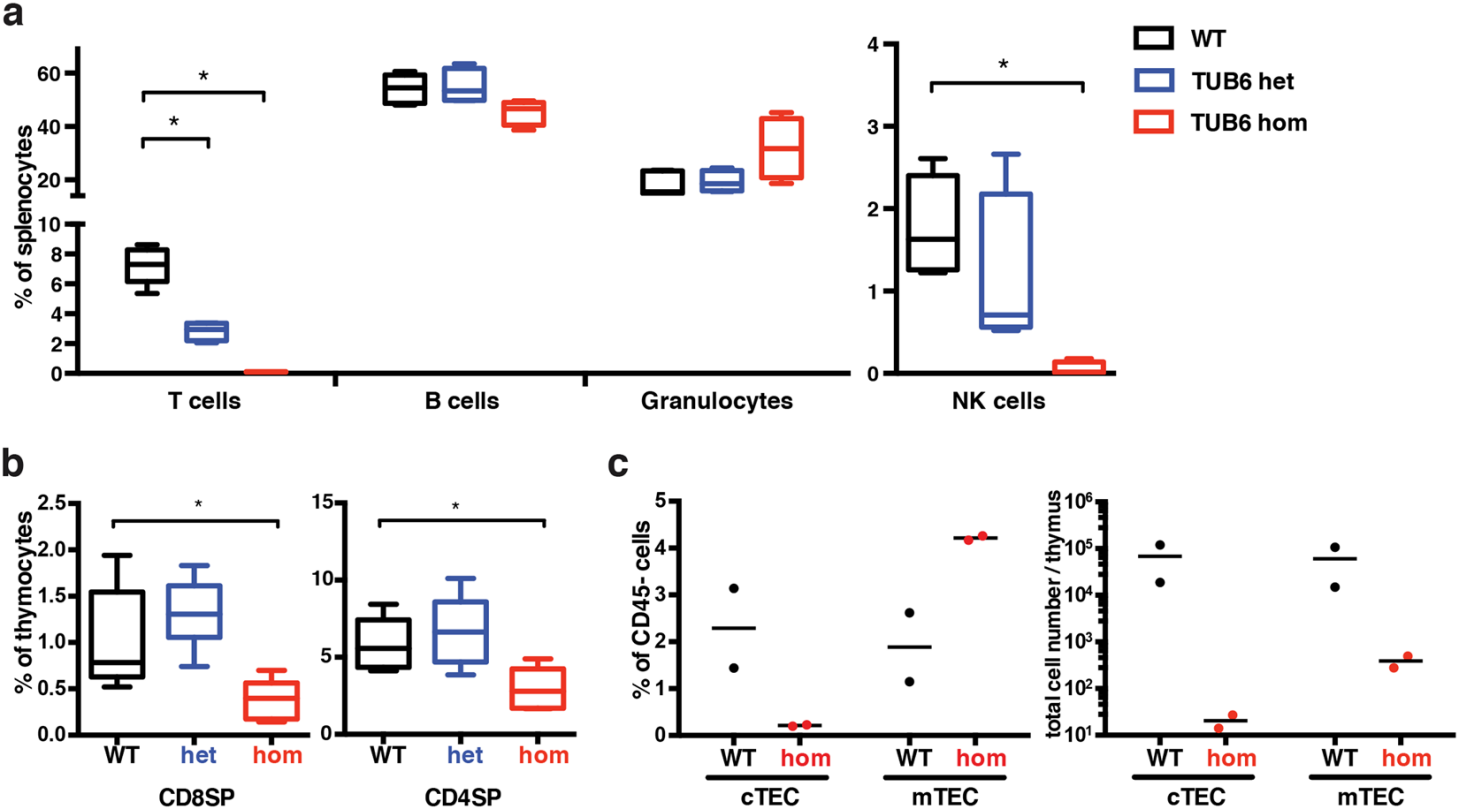
Phenotype of neonatal TUB6 mutants. Flow cytometric analysis of indicated cell populations from 2–5-day-old pups: splenocyte frequencies (**a**) and CD8SP and CD4SP thymocytes (**b**). a and b show pooled data from 3 litters analysed on 3 different days (n = 5 WT, 4 het, 4 hom). (**c**) Analysis of thymic stroma: cortical thymic epithelial cells (cTECs: Epcam^+^Ly51^+^) and medullary thymic epithelial cells (mTECs: Epcam^+^Ly51^−^), gated on CD45^−^ cells (n = 2), shown as percentages of CD45^−^ cells or as total cell counts. (**a**–**c**) For flow cytometry dot plots see Supplementary Fig. S3.

In contrast to athymic adolescent homozygotes, vestigial thymi were present in new-born homozygotes, excluding a general thymus anlage defect. Despite the presence of a thymus, T cells were profoundly reduced in peripheral blood and spleens from homozygous TUB6 neonates (Fig. 3a). NK cells were also drastically decreased in the periphery, whereas B cell and granulocyte frequencies were comparable to wild type levels (Fig. 3a). Flow cytometric analysis of TUB6 homozygous neonatal thymi revealed decreased frequencies of CD4SP (single positive) and CD8SP thymocytes compared to control littermates (Fig. 3b), suggesting arrest during thymocyte development at the double positive (DP) stage. Furthermore, flow cytometric analysis of thymic stroma cells revealed lack of cTECs in thymi from homozygous neonates (Fig. 3c).

We hypothesised that vanishing of the B cell- and simultaneous expansion of the granulocyte-compartment might be due to enhanced susceptibility to opportunistic infection(s) as a consequence of the inborn severe T and NK cell deficiency. To test this hypothesis, we evaluated the TUB6 mutant phenotype after transfer of the line into a germ-free environment. However, the disease developed identically under germ-free conditions, and germ-free heterozygous as well as homozygous TUB6 mice represented phenocopies of mutants with a conventional microflora (Supplementary Fig. S3), including homozygous lethality. These findings indicate that the TUB6 phenotype results from sterile inflammation and develops independently of the microbiome.

### Underlying genetic alteration identified as a point mutation in Psmb10, encoding MECL-1

In an initial mapping attempt to identify the chromosomal localisation of the underlying genetic mutation, we found a strong linkage with markers located on chromosome 8 (Supplementary Fig. S4). The strongest linkage (logarithm of the odds (LOD) >16) was detected with rs13479998 (8: 114,166 Mb, http://ensembl.org), although the mapping approach still left a large candidate region. Comparison of the TUB6 phenotype with existing gene-targeted mice and human inherited disorders failed to reduce the number of candidate genes.

**Figure 4:**
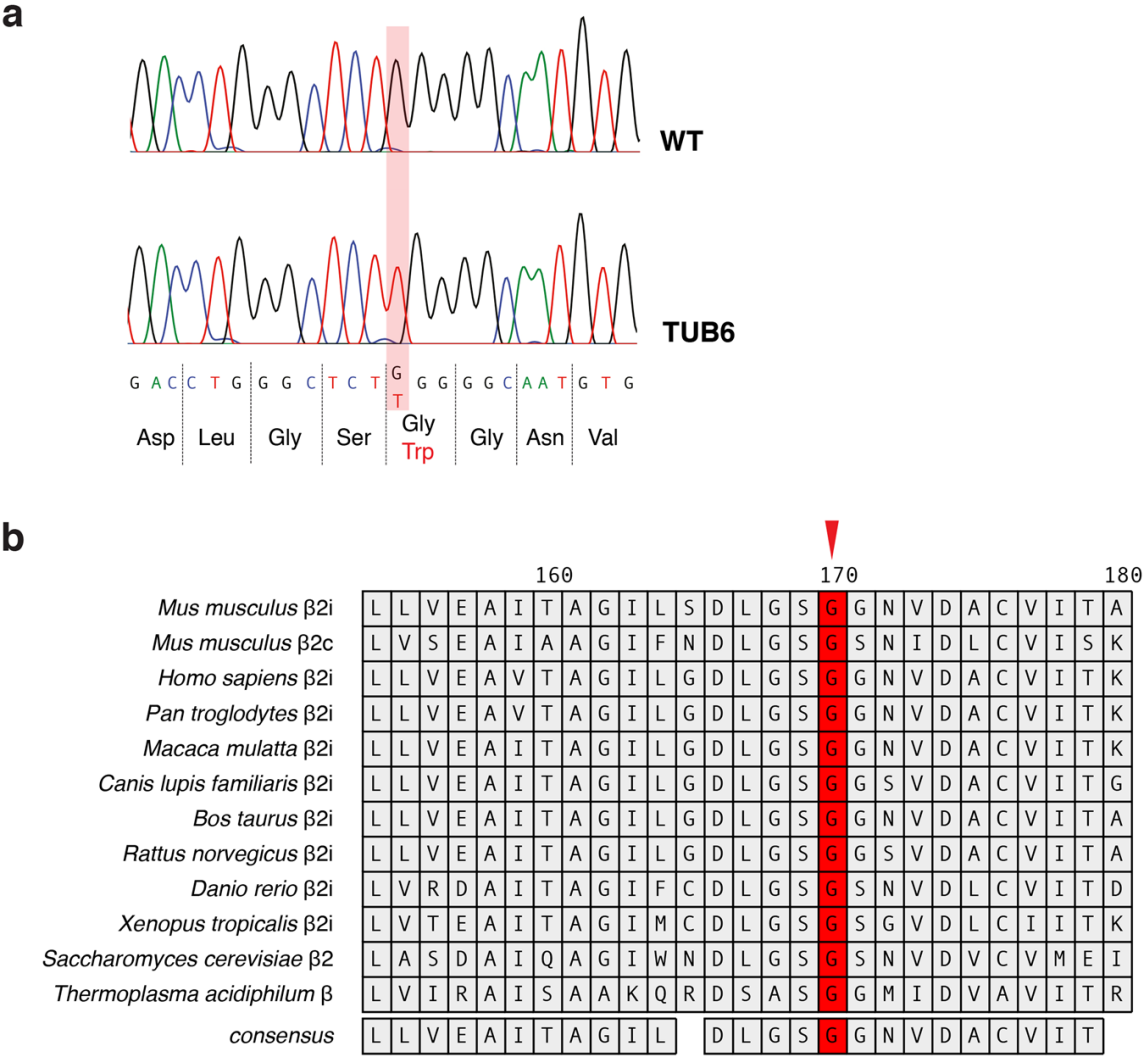
Underlying genetic alteration identified as missense point mutation in Psmb10 at a highly conserved position. (**a**) Sanger sequencing of the *Psmb10* gene exon 7 shows the G > T nucleotide substitution leading to an amino acid exchange from Gly to Trp in TUB6 mice. (**b**) Multi-species alignment revealed the strict evolutionary conservation of glycine 170 (indicated by a red arrow). Amino acid numbers are assigned according to the sequence alignment to the β-subunit of *Thermoplasma acidophilum*.

The vast majority of ENU-induced genetic alterations are based on critical point mutations in coding regions or splice sites, therefore we utilised deep whole exome sequencing to identify the underlying TUB6 mutation^37,38^. We found only one homozygous point mutation present in analysed affected mice but not in the wild type controls (Fig. 4a). This mutation was located exactly within the previously mapped candidate region.

The mutated gene *Psmb10* encodes MECL-1, one of three catalytic subunits of the iCP and tCP. The identified point mutation leads to an amino acid exchange from glycine to tryptophan at the strictly conserved position 170 (Fig. 4b). In order to validate the mutation, we analysed tail DNA samples from more than 500 inbred mice and found a phenotype-genotype correlation of 100%. Furthermore, we backcrossed the TUB6 mouse line onto C57BL/6 (F7) and BALB/c (F11) backgrounds and found strong phenotype persistence (Supplementary Fig. S4). When TUB6 mice were crossed to MECL-1 KO mice, the resulting hemizygous offspring (MECL-1^−/G170W^) presented all key features of the homozygous TUB6 phenotype, i.e. lack of T and NK cells and substantial lethality (leukocyte frequencies are shown in Supplementary Fig. S4). These findings demonstrate the causal relationship of the MECL-1^G170W^ mutation and the severe TUB6 phenotype.

### Overexpression of MECL-1^G170W^ is lethal for murine cells

**Figure 5:**
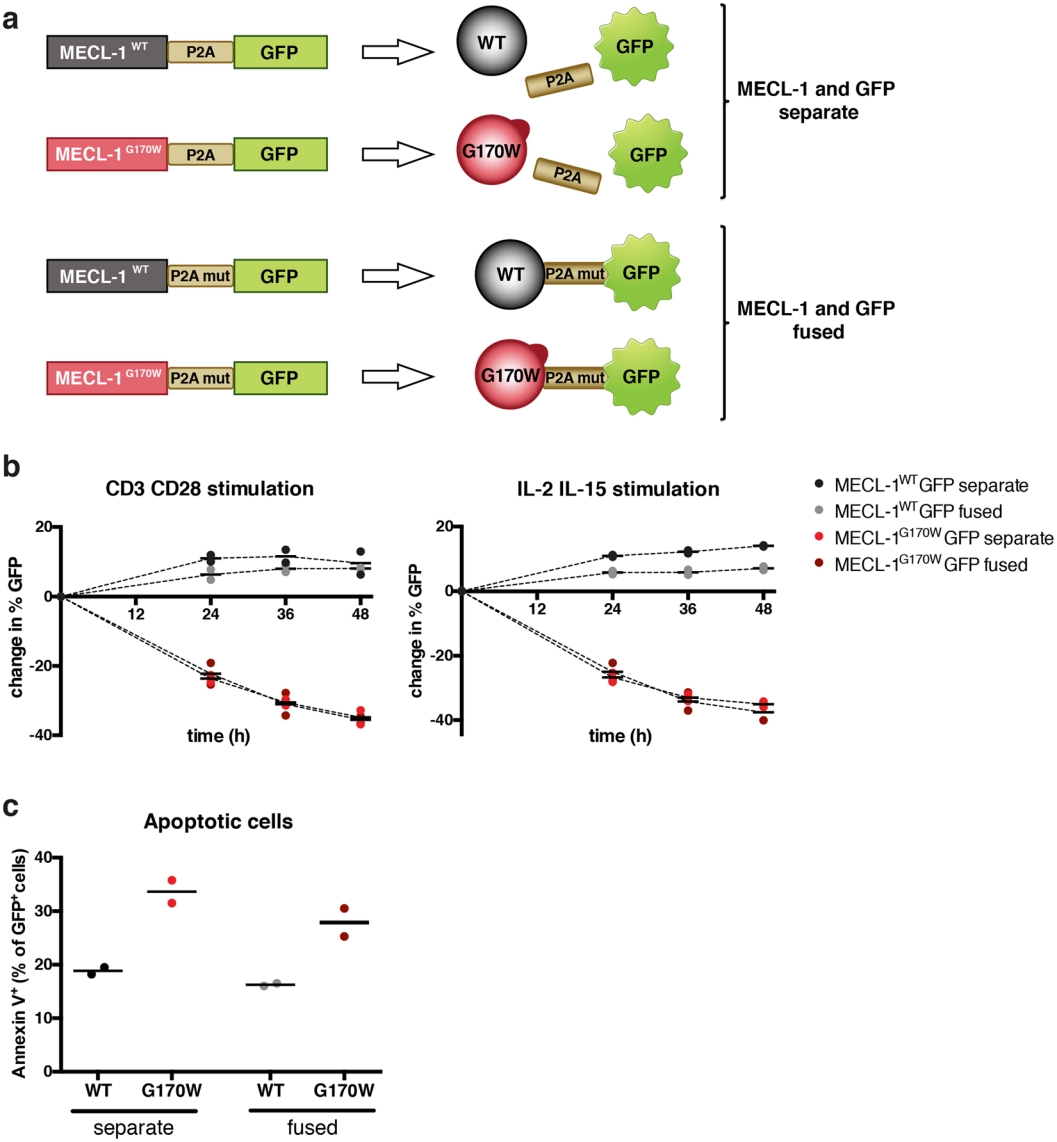
Overexpression of mutated MECL-1 causes cell death in murine splenocytes. (**a**) Schematic illustration of the GFP (green fluorescent protein) reporter constructs used for transduction. Fusion of MECL-1 and GFP ensures co-expression of both proteins. To rule out fusion effects, two different linkers between MECL-1 and GFP were used: In the first setting the P2A self-cleavage motif hydrolyses the protein chain, resulting in two separate proteins. In the second setting the self-cleavage ability of P2A was abrogated by a mutation, leading to expression of one fused MECL-1 GFP protein. (**b**) Kinetics of the GFP signal detected in the splenocyte population after transduction. Plots show changes in GFP signal relative to transduction efficacy. After transduction, T cells were stimulated with anti-CD3 and anti-CD28 antibodies (left panel) or IL-2 and IL-15 (right panel). (**c**) Apoptotic cells identified by Annexin V staining 36 h after transduction are plotted as percentages of transduced (GFP^+^) cells. Data from one representative overexpression experiment are shown, in total the experiment was performed twice with similar results. In each experiment splenocytes were transduced with two different virus supernatants. Each point in the dot plots shows data from one separately transduced splenocyte sample.

To assess the effect of MECL-1^G170W^ on murine cells, we cloned the coding sequences of wild type MECL-1 and MECL-1^G170W^ into the retroviral mP71 vector. The generated constructs (Fig. 5a) were used to produce viral particles for transduction of primary wild type splenocytes. Due to the fact that retroviruses infect only proliferating cells, splenocytes were stimulated with agonistic anti-CD3 and anti-CD28 antibodies to induce T cell proliferation 24 h prior to transduction. Following retroviral transduction, two parallel culture conditions for splenocytes were applied: either prolonged stimulation with anti-CD3 and anti-CD28 antibodies, or incubation with interleukin (IL)-2 and IL-15. The frequency of green fluorescent protein (GFP)^+^ cells decreased over time in cells transduced with MECL-1^G170W^ (Fig. 5b), showing a lethal effect of MECL-1^G170W^ overexpression even in presence of endogenous wild type MECL-1. Furthermore, there was no evidence for stimulus-dependent effects. Both, the T cell receptor (TCR)-engaging CD3/CD28 and TCR-bypassing IL-2/IL15 stimulations lead to a similar decrease of MECL-1^G170W^-expressing cells (Fig. 5b). Annexin V staining 36 h post transduction revealed higher frequencies of apoptotic cells, showing an increase of programmed cell death in MECL-1^G170W^ expressing cells (Fig. 5c). Taken together, these results clearly demonstrate lethal effects of MECL-1^G170W^ overexpression on murine splenocytes. Furthermore, MECL-1^G170W^ transduced T cells undergo rapid apoptosis independent of persistent TCR engagement.

### G170W mutation in analogous yeast β2 subunit is lethal

**Figure 6:**
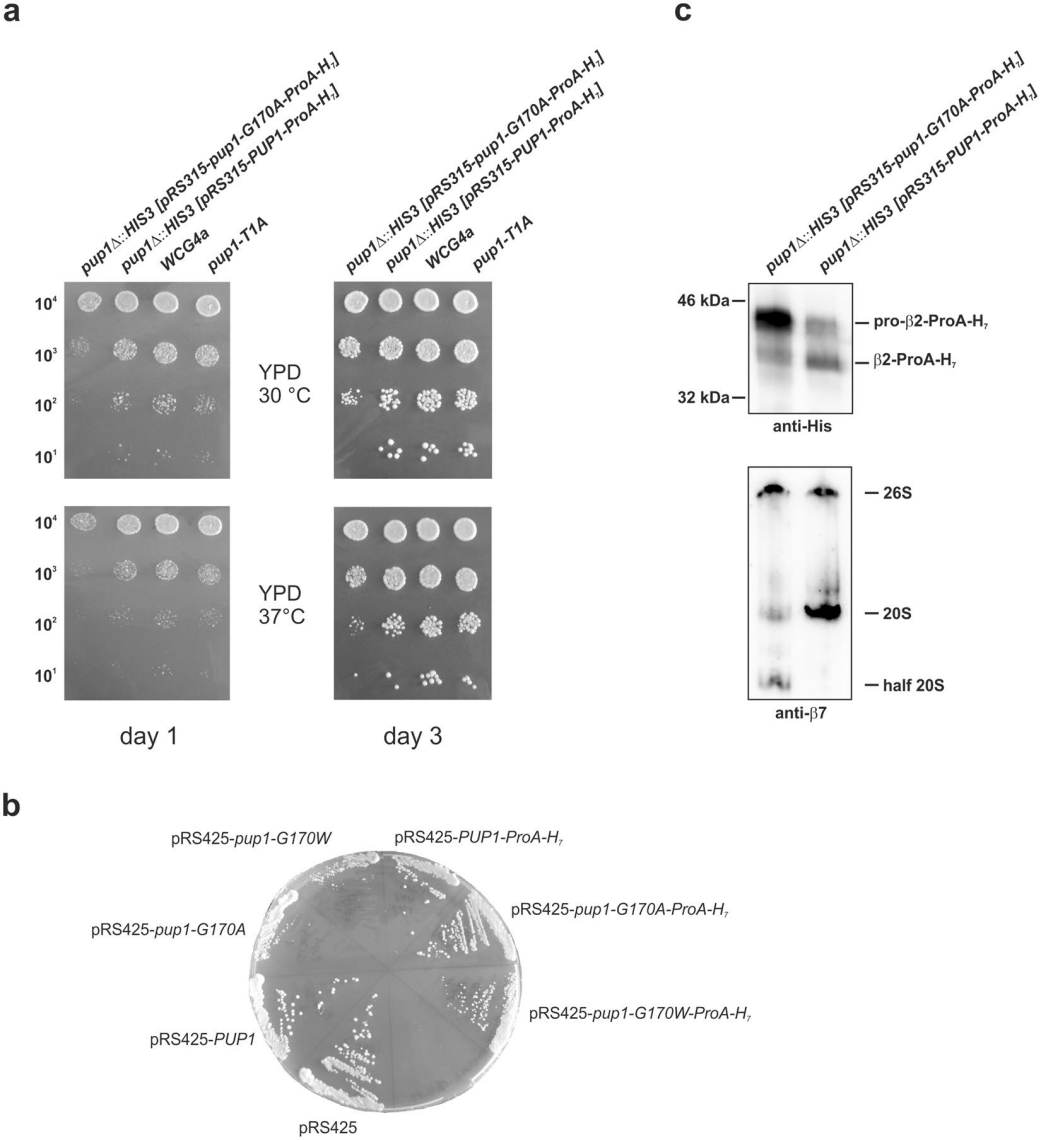
Yeast mutagenesis experiments. (**a**) Drop test of mutant yeast strains. Indicated cell numbers were spotted on YPD plates and incubated for 1–3 days either at 30 or 37 °C. Growth of the yeast β2 (*pup1*) G170A-ProA-H_7_ mutant is retarded compared to the respective wild type strains and compared to the β2 loss of function mutant T1A. (**b**) Overexpression of mutant yeast β2-subunits. Wild type yeast (WCG4a) was transformed by either the empty 2 µ plasmid pRS425 or variants thereof encoding wild type *PUP1* or mutant *pup1* genes. Yeasts were streaked on CM Leu^−^ plates (to select for the plasmid pRS425) and grown at 30 °C. (**c**) Western blot of equal amounts of β2 (*pup1*) G170A-ProA-H_7_ and β2 (*PUP1*)-ProA-H_7_ cell lysates separated by SDS-PAGE (upper panel) or native PAGE (lower panel). Anti-His immunoblotting of denatured lysates reveals that the mutant accumulates immature β2 (pro-β2), while in wild type cells the processed version of β2 is more abundant. In agreement, probing native lysates with an anti-β7 antibody visualises the accumulation of half 20S proteasomes in the mutant. Note, β7 is the last proteasome subunit that is incorporated into half proteasome precursors. Full-length western blots are presented in Supplementary Fig. S6.

Gly170 is located in a structurally highly conserved region of subunit β2 at the interface of the two proteasome β-rings in both the mouse iCP^3^ and the yCP^39^. Hence, its mutation to Trp was assumed to sterically hinder dimerisation of two half-CPs and to interfere with CP assembly. To prove this hypothesis, we conducted yeast mutagenesis experiments. Noteworthy, yeast expresses only one type of CP and any interruption of its assembly is lethal. We introduced the TUB6 point mutation G170W into the *LEU2*-plasmid pRS315-*PUP1*, encoding the β2 subunit of the yCP. Transformation of the yeast strain YWH10 (*pup1Δ*::*HIS3* [pRS316-*PUP1*]) with the constructed mutant (mt) plasmid and subsequent selection of transformants on 5-FOA plates for removal of the URA3-marked pRS316-plasmid encoding wild type (WT) *PUP1* demonstrated that the *pup1*^*G170W*^ mutant was not viable. Because expression of a proteolytically inactive *pup1* variant (e.g. Thr1Ala) does not provoke any phenotype in yeast^40,41^ a general proteasome assembly defect was supposed to cause the lethality of *pup1*^G170W^ (Fig. 6a).

In parallel, we performed the plasmid shuffling with a construct encoding Ala on the same position 170. Like *pup1*^*G170W*^, the *pup1*^*G170A*^ mutation was lethal to yeast, yet viability could be restored by fusing a Protein A-His_7_-tag (ProA-H_7_) to the C-terminus of the mutant yβ2^G170A^ subunit. Although the resulting *pup1*^*G170A*^*-ProA-H*_7_ mutant suffered from a pronounced growth defect (Fig. 6a), its viability was indicative of the successful incorporation of the mutant yβ2 subunit into yCPs. Notably, proteasome activity in the *pup1*^*G170A*^*-ProA-H*_7_ mutant cells was not impaired compared to wild type strains - a fact that might arise from proteasome subunit overexpression to overcome an assembly defect (Supplementary Fig. S5). In contrast to the *pup1*^*G1*7*0A*^ mutant, a C-terminal ProA-His_7_-tag could not rescue the *pup1*^*G1*7*0W*^ yeasts. These results suggest that the bulky tryptophan residue has additional negative effects on the proteasome assembly line compared to the smaller alanine.

Next, the mutant alleles were overexpressed in WT yeast, using a 2 µ plasmid. Consistent with the shuffling experiments, we observed clearly reduced growth rates and colony sizes of the *pup1*^*G1*7*0A*^, *pup1*^*G1*7*0W*^ and *pup1*^*G1*7*0W*^*-ProA-H*_*7*_ transformants compared to the controls and compared to the *pup1*^*G170A*^*-ProA-H*_*7*_ expressing yeasts (Fig. 6b). Although the *ProA-H*_*7*_ tag has a positive effect on the proliferation of WT yeasts that overexpress the G170A and G170W mutant alleles, this partial rescue is insufficient to overcome lethality of the *pup1*^*G170W*^*-ProA-H*_*7*_ mutant during plasmid shuffling.

Proteasome assembly defects, as supposed for the *pup1*^*G170A*^*(-ProA-H*_*7*_) *and pup1*^*G1*7*0W*^*(-ProA-H*_*7*_) mutants, often lead to reduced proteasome activity because immature intermediates as well as inactive β subunits with propeptides attached (proproteins) accumulate. We therefore analysed lysates of *pup1*^*G1*7*0A*^*-ProA-H*_7_ mutant and *PUP1-ProA-H*_7_ wild type yeast cells by SDS- or native PAGE, followed by immunoblotting against either the His-tag or yβ7, the endmost subunit incorporated into half 20S proteasomes. These experiments clearly showed that yβ2-precursor subunits as well as half yCPs accumulate in the mutant (Fig. 6c and Supplementary Fig. S6).

In conclusion, mutation of yβ2-Gly170 severely disturbs the proteasome assembly line. Lethality of a G170A mutation can be rescued by a C-terminal ProA-H_7_-tag, but in agreement with the growth defect observed, proteasome maturation is impaired and half CPs as well as unprocessed yβ2 subunits accumulate, suggesting that dimerisation of half CPs is sterically hindered in the *pup1*^*G1*7*0A*^*-ProA-H*_7_ mutant.

### G170A mutation distorts the assembly-relevant C-terminal appendage of subunit β2

The *pup1*^*G1*7*0A*^*-ProA-H*_7_ mutant yCP was purified by affinity chromatography and crystallised after tag removal by Tobacco Etch Virus (TEV) protease (Supplementary Fig. S7). The structural data (Supplementary Table S1) visualised the incorporation of the point mutation G170A and verified correct autolysis of the yβ2-propeptide. Furthermore, a crystal structure in complex with the inhibitor bortezomib (Velcade^®^) displayed full occupancy of the ligand at each proteolytic centre, thereby confirming the functional integrity and reactivity of all proteasomal active sites (Supplementary Fig. S8). Owing to the purification procedure, which is based on activity assays to identify proteasome-containing fractions, only active particles but no assembly intermediates were isolated (see Materials and Methods).

**Figure 7:**
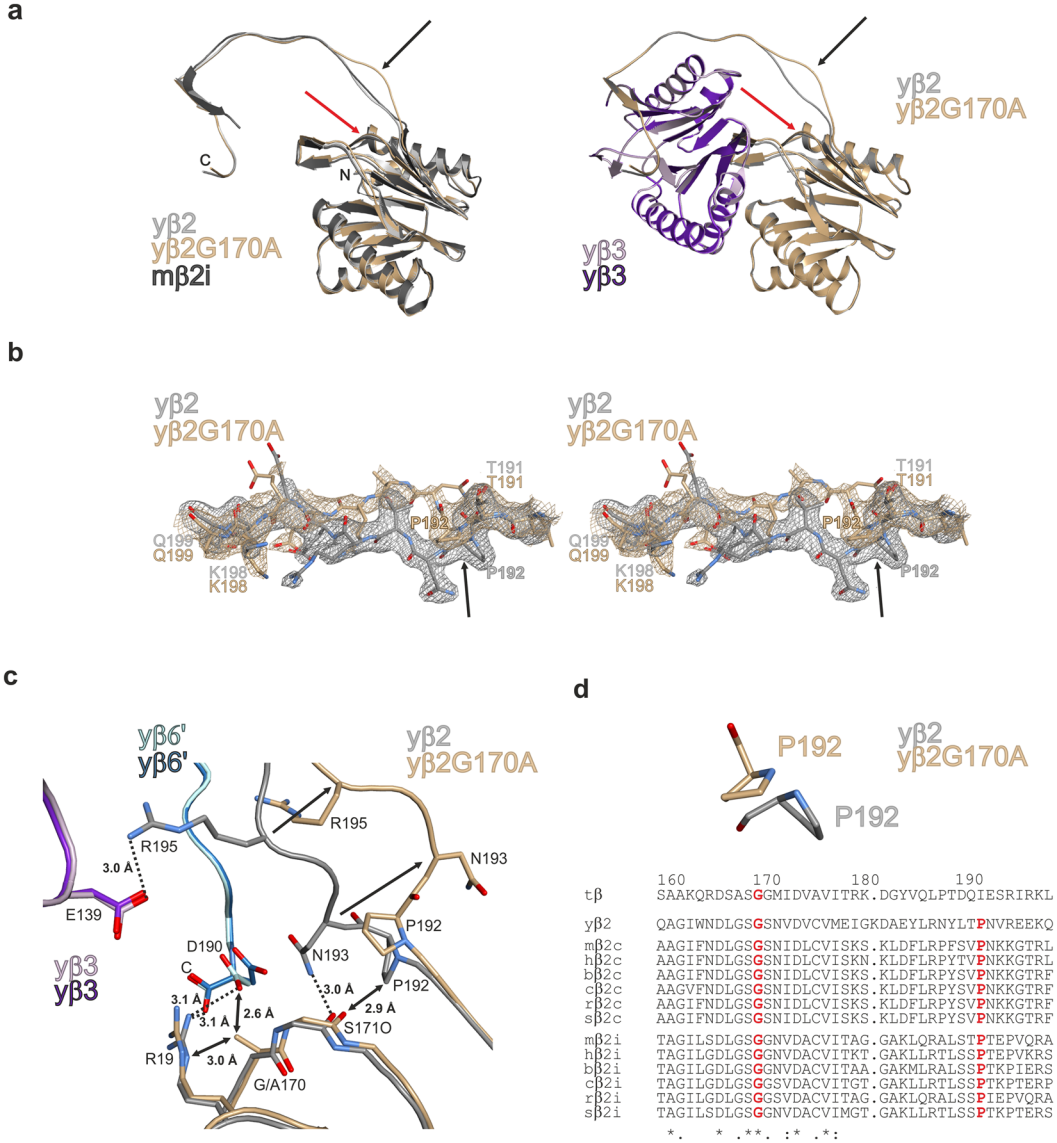
Crystal structure of the yeast β2-G170A mutant proteasome. (**a**) Ribbon illustration of the G170A mutant yeast proteasome subunit β2 (brown) superimposed onto the wild type counterpart (grey) and the mouse iCP subunit β2i (black) on the left; association of the subunits β2 and β3 (purple) is shown on the right. The site of mutation and the resulting conformational changes in the β2 C-terminal tail are marked by red and black arrows, respectively. (**b**) Stereo representation of the 2F_O_-F_C_ omit electron density maps for the G170A mutant (brownish) and wild type yβ2 subunit (grey) contoured to 1σ. Note the structural changes and the increased flexibility of the loop segment in the mutant structure. The flip of Pro192 is marked by a black arrow. (**c**) Hydrogen-bonding network around the site of mutation. Exchange of Gly170 in subunit yβ2 results in the reorientation of residues 192–196 (black arrows). Hereby hydrogen bonds (black dotted lines) within yβ2 and with the neighbouring subunits yβ3 and yβ6′ (of the opposite half proteasome) are broken. Steric clashes are marked by black double arrows. (**d**) The strictly conserved Pro192 is supposed to orient the β2 C-terminus for association with the adjacent β3 subunit. In the mutant crystal structure, flipping of Pro192 changes the conformation of the β2 C-terminus. Species abbreviation in sequence alignment: t: *T*. *acidophilum*; y: yeast; m: mouse; h: human; b: *Bos taurus*; c: *Canis familiaris*; r: *Rattus norvegicus*; s: *Sus scrofa*; amino acid numbers are assigned according to the β-subunit of *T*. *acidophilum*.

The structural data visualise that WT and mutant yβ2-subunits superimpose well (WT/mt: r.m.s.d. 0.3 Å), except for the segment Pro192-Glu197. In the mutant structure this loop is strongly distorted and less well resolved in the 2F_O_-F_C_ electron density map compared to the WT yβ2-subunit (Fig. 7a,b). Detailed inspection of the site of mutation reveals that Ala170 would be too close to Arg19 from the same subunit (3.0 Å) as well as to the C-terminal amino acid Asp190 from subunit yβ6′ of the opposite β-ring (2.6 Å; Fig. 7c). To avoid steric clashes, the guanidine group of Arg19 (yβ2) and the carboxylate of Asp190 (yβ6′) are shifted by up to 1.6 Å. Hereby, the hydrogen bonds between Arg19NH2 and Asp190OD2 (WT: 3.1 Å; mt: 5.1 Å) as well as Arg19NH2 and the C-terminus of subunit yβ6 (WT: 3.1 Å; mt: 4.5 Å) are broken. Furthermore, slight backbone distortions around the mutation shorten the distance between Ser171O and Pro192CD from 3.5 Å to 2.9 Å. This close contact leads to a flip of Pro192 out of the hydrophobic pocket provided by Ile163, Trp164, Val173 and Leu190 of yβ2 (Fig. 7c), however, without changing its trans conformation (Fig. 7d). The reorientation of Pro192 triggers twisting of the succeeding yβ2-amino acids Asn193-Glu196 outwards on the protein surface (shift of Pro192C^α^: 1.6 Å; Asn193C^α^: 6.6 Å; Val194C^α^: 5.7 Å; Arg195C^α^: 4.8 Å; Glu196C^α^: 2.0 Å; Glu197C^α^: 0.6 Å; Fig. 7c), hereby loosening intra- and inter-subunit contacts. For instance, the hydrogen bridges between Asn193ND2 and Ser171O (WT: 3.0 Å; mt: 9.6 Å) as well as Arg195NH2 and Glu139OE2 of yβ3 (WT: 3.0 Å; mt: 11.7 Å) are broken. All these structural changes affect the orientation of the C-terminal appendage of subunit β2, which is vital for eukaryotic half-CP assembly by governing the association with the neighbouring subunit β3 via β-sheet interactions^28,38^. Most likely, mutation of Gly170 enhances the structural flexibility of the C-terminal tail of yβ2 and impairs the association with subunit yβ3 of the same β-ring/CP half (*cis* interactions). The very C-terminus of subunit β2 approaches subunit α3 of the same CP half, suggesting that the ProA-tag facilitates *cis* interactions (within the same hemiproteasome) and thereby compensates for the deleterious conformational change in the yβ2-appendage. Besides, mutation of Gly170 appears to impair half-proteasome association for steric reasons (Fig. 6c).

Western blotting of spleen and thymus lysates from homozygous TUB6 mutants revealed an accumulation of the MECL-1^G170W^ precursor and reduction of processed MECL-1^G170W^ (Supplementary Fig. S9). Furthermore, in lysates of primary mouse embryonic fibroblasts derived from mutant TUB6 mice, a partial accumulation of the MECL-1 precursor was observed (Supplementary Fig. S9), supporting the conclusion that mutation of Gly170 in the proteasomal β2 subunit induces pronounced structural changes that impair proper particle assembly.

## Discussion

Here we describe for the first time the mutant mouse line TUB6 that carries the point mutation G170W in MECL-1, the β2i subunit of the iCP and tCP. Heterozygous mutants are characterised by reduced numbers of T cells at baseline conditions and an impaired T cell response to *Listeria* challenge. While the early innate response to *Listeria* involving neutrophil granulocytes, NK cells, and macrophages is able to control the infection in the first 3 days^42^, TUB6 mutants succumb later during the course of infection, when *Listeria*-specific IFN-γ-producing CD8^+^ effector T cells are indispensable for bacterial clearance^43,44^. However, PMA/ionomycin stimulation demonstrated that MECL-1^+/G170W^ T cells are still capable of producing effector cytokines, ruling out a general T cell activation defect.

The homozygous TUB6 phenotype matches the main symptoms of the human diseases ALDD/PRAAS, i.e. autoinflammation, granulocyte infiltration into the skin, and lipodystrophy^19–21,23^. In addition, TUB6 mice develop B^-^T^-^NK^-^SCID, which was not reported in ALDD/PRAAS patients so far. MECL-1 is part of both the iCP and tCP. Deficiency for tCPs alters the positive selection of CD8^+^ T cells, thereby reducing CD8SP thymocytes and CD8^+^ T cells in the periphery, but not CD4SP thymocytes and CD4^+^ T cells^12,13^. The absence of CD4SP thymocytes and peripheral CD4^+^ T cells in homozygous TUB6 mice compared to the β5t^−/−^ KO might result from the complete lack of the tCP-expressing cTECs. This is supported by the finding that transient cTEC depletion leads to impaired development of both, CD4^+^ and CD8^+^ T cells^45^. Since other cell types such as NK- and B-cells are unaffected by cTEC depletion, additional factors are assumed to cause SCID in TUB6. The B cell loss in TUB6 temporally correlates with granulocyte expansion and may be based on exposure and enhanced susceptibility to opportunistic infections. Accelerated granulocyte production in response to infection is known as “emergency granulopoiesis” and is accompanied by decreased lymphocyte production^46^. The TUB6 mutant, however, develops this disease also under germ-free breeding conditions, indicating establishment of SCID and sterile autoinflammation in the absence of microbial triggers.

**Figure 8:**
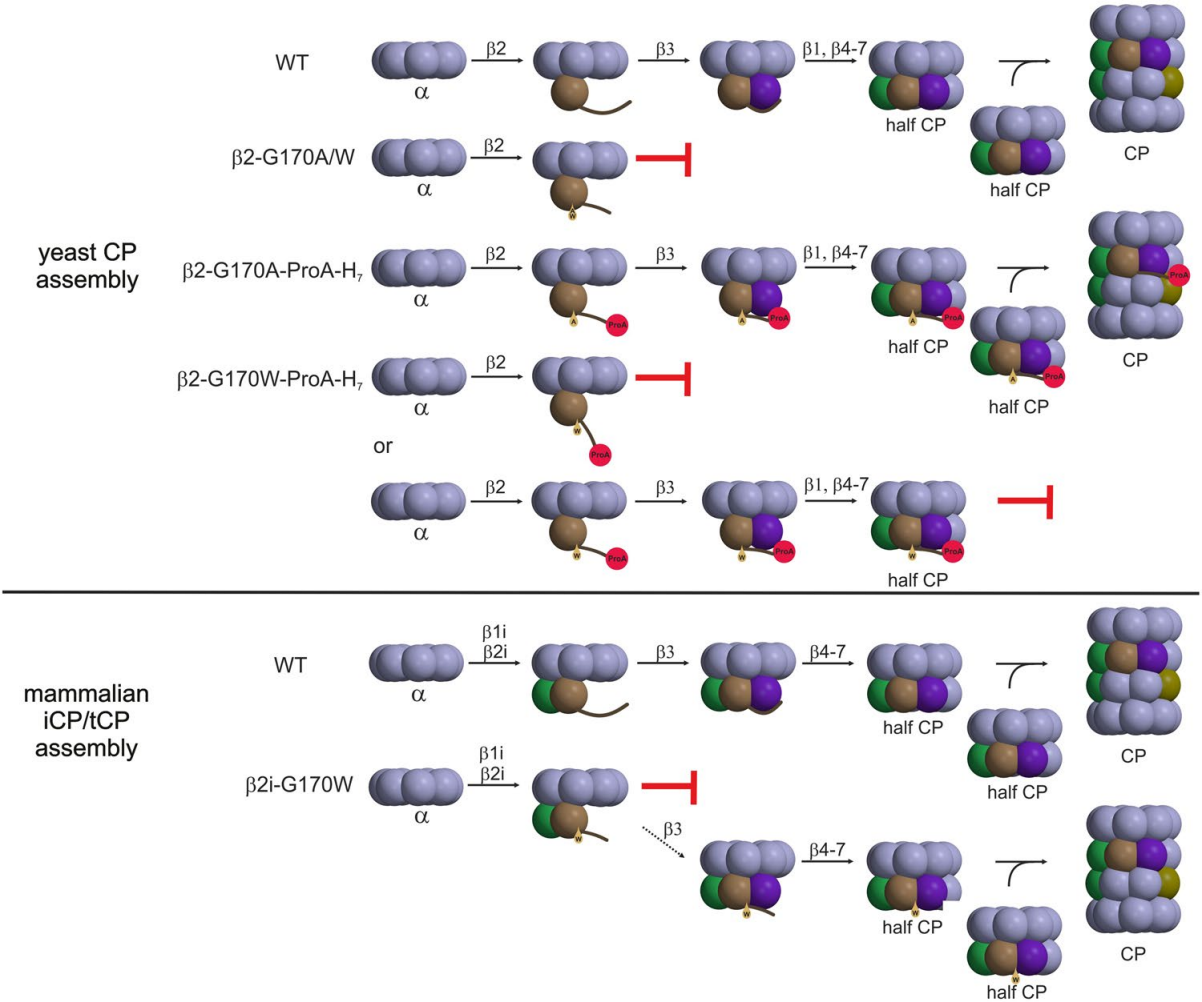
Model of CP assembly and proposed impact of the TUB6 mutation on iCP/tCP biogenesis. The mutations G170W and G170A in the yβ2 proteasome subunit abrogate assembly of the yeast CP (upper panel). Since the β2-G170A-Pro-H_7_ mutant accumulates half CPs (Fig. 6c), the β2-G170W-Pro-H_7_ mutant is likely to suffer from a defect concerning half CP dimerisation. Similarly, the TUB6 mutation G170W in the proteasome subunit β2i leads to a major arrest in precursor complex processing and an inefficient, minor progression to mature iCP/tCP (lower panel). Colour code: β1 (green), β2 (brown), β3 (violet), β5 (olive).

In order to improve our understanding of the pathogenesis in TUB6, we were interested in elucidating its molecular basis. Mutagenesis experiments in yeast, expressing only one CP variant, revealed that any change of Gly170 in the yeast proteasome subunit yβ2, to either Trp or Ala, is lethal. Since inactivation of the yβ2 active site does not interfere with yeast viability^40,41^, the mutation of Gly170 was supposed to hamper CP biogenesis. Fortunately, fusion of a ProA-H_7_-tag to the yeast β2-C-terminus rescued the G170A mutant and enabled the structural analysis of its yCP. The X-ray crystallographic data visualise that the point mutation G170A leads to significant rearrangements in a loop region of the C-terminal appendage of subunit yβ2. The C-terminal extension of β2 is pivotal for survival of yeast and mammalian cells, as it governs the incorporation of the adjacent subunit yβ3^28,29^. The very C-terminal β-sheet, engaged in interactions with the neighbouring subunit yβ3 is unaffected in the mutant; however, the unusual arrangement of the yβ2 tail may interfere with yβ3 association and thus cause failure of the proteasome assembly line in the *pup1*^*G1*7*0A*^ and *pup1*^*G1*7*0W*^ mutants (Fig. 8). Presumably, fusion of the C-terminal ProA-H_7_-tag to yβ2 stabilises the interactions of yβ2 with yβ3 and with the α-ring, thereby supporting half-proteasome formation in the *pup1*^*G1*7*0A*^*-ProA-H*_7_ strain. Nonetheless, dimerisation of the half-CPs seems to be impaired (Fig. 6c). In contrast to the *pup1*^*G1*7*0A*^ yeasts, the mutation G170W is lethal in the absence and presence of the ProA-H_7_-tag, implying that the bulky tryptophan either completely blocks β-ring assembly or prevents fusion of hemiproteasomes for steric reasons. Considering that the *pup1*^*G170A*^*-ProA-H*_*7*_ mutant accumulates half CPs (Fig. 6c), the latter hypothesis likely applies to the *pup1*^*G170W*^*-ProA-H*_*7*_ mutant (Fig. 8).

Notably, the severe immunological defect of the TUB6 mouse clearly contrasts the mild phenotypes of the reported KO mice lacking iCP or tCP subunits^8,9,11–13^, but our following model provides an explanation: In MECL-1 deficient mice the corresponding constitutive subunit β2c fills in for MECL-1 and thus, CP assembly leads to either homogenous cCPs or mixed type CPs containing the subunits β2c, β1i, β5i or β2c, β1c, β5i^14^. Similarly, β5t KO mice are assumed to incorporate either β5c or β5i in their CPs as a mechanism of compensation^47^ and iCP triple KO mice can at least assemble CPs containing β2c, β1c, β5c as well as β2c, β1c, β5t in the absence of the immunosubunits. Furthermore, due to the availability of subunit β5c, also β5t^−/−^ β5i^−/−^ mice do not suffer from a phenotype comparable to the TUB6 mouse^13^. All these KO mice still can form at least one type of functional CP that ensures cell survival, thus the resulting phenotypes are modest and manifest in antigen presentation defects leading to reduced CD8^+^ T cell repertoires^8,11,13^.

By contrast, the mutant MECL-1^G170W^ cannot be substituted by its constitutive counterpart β2c, because in cells expressing immuno- and constitutive subunits association of the heptameric α-ring with MECL-1 and β1i is preferred over β2c incorporation^48^ (Fig. 8). Our structural data on the β2-G170A mutant yCP suggest that the mutation G170W in MECL-1 does not alter the interactions with the α-ring, but may prevent or destabilise the association with subunit β3, thereby leading to the formation of dead-end CP intermediates, containing the α-ring, β1i and MECL-1^G170W^.

In agreement, our data show that overexpression of MECL-1^G170W^ is lethal for murine T cells and that the immature MECL-1 precursor is found in the thymus and the spleen of homozygous TUB6 mutants. The failure to produce enough functional CP in MECL-1^G170W^-expressing cells ultimately results in their massive demise and immunodeficiency. Cell death is a potent trigger of sterile inflammation marked by neutrophil recruitment^49^. Thus, this model also explains the observed granulocytosis and neutrophil infiltration into tissues.

Reminiscent of the TUB6 mouse, substantial loss of cTECs and thymic tissue was recently reported for a Gly170Arg missense mutation in *Psmb11*, the gene encoding the tCP-specific subunit β5t^50^. Interestingly, subunit β5t features an extended C-terminus similar to β2 subunits, which is absent in β5c and β5i^12^. The β5t^G170R^ mutant subunit was found to be immature, to induce the accumulation of tCP precursor complexes, and to trigger cell death in β5t-expressing cells^50^. Due to the lack of any structural information on the tCP, it still remains to be elucidated which of the β subunits fails to interact with the mutant β5t subunit. The distinct phenotypes of the β5t and β2i missense mutations versus the respective KOs, however, demonstrate the power of phenotype-driven “forward genetics” to identify a broader range of gene functions than with the “reverse” gene-driven approach^51^. Besides the insights into basic proteasome biology, TUB6 provides the first mouse model for human ALDD/PRAAS syndromes. Although for human PRAAS a global proteasome dysfunction is suggested, rather than specific immunoproteasome dysfunction^23^, the TUB6 phenotype is associated to defects in immuno- and/or thymoproteasome biology, particularly regarding the early T cell loss in new-born homozygotes and also the heterozygous T cell-specific phenotype.

Given the high structural and functional homology of the murine and human MECL-1, and the severe infection susceptibility phenotype in heterozygotes, our data suggest to include MECL-1 as a candidate gene for screening in human individuals with primary immunodeficiency of unknown aetiology.

## Methods

### General statistical evaluation

Box plots indicate the median, the first and third quartiles, and the minimum and maximum values. Statistical differences between groups were determined by the Mann-Whitney rank-sum using Prism v.6 (GraphPad Software, Inc., La Jolla, CA). Asterisks indicate P values *<0.05, **<0.01, ***<0.001, ****<0.0001.

### Mice

All mouse experiments were approved by the Regierung of Oberbayern and all methods were carried out in accordance with the approved guidelines.

The TUB6 mutation was generated on the inbred C3HeB/FeJ strain purchased originally from the Jackson Laboratory (Bar Harbor, ME, USA) in a large-scale ENU mouse mutagenesis program as previously described^31,52,53^. Mice were housed under specific pathogen-free conditions at the mouse facility at the German Mouse Clinic.

Mice were originally tentatively grouped into wild type, heterozygous and homozygous groups according to their phenotype. Genotyping was performed retrospectively after the mutation was found.

For generation of germ-free TUB6 mice, 4–6-week-old heterozygous TUB6 females were superovulated by intraperitoneal (i.p.) injection of 7.5 international unit (IU) pregnant mare serum gonadotropin (PMSG), and 4 h later 7.5 IU human chorionic gonadotropin (hCG), and on the same evening paired with heterozygous TUB6 males. On the following morning, successful mating was controlled by plug check; plug positive females were shipped to Clean Mouse Facility (CMF), University of Bern (Bern, Switzerland). Upon arrival, the females were euthanised and the transfer of two-cell embryos into pseudopregnant female recipients was performed as described previously^54^.

### Infection

Infection experiments were performed with the wild type *L*.*m*. strain 10403s. Brain heart infusion (BHI) medium was inoculated with *Listeria* stock solution and incubated at 37 °C until an OD_600_ of 0.05–0.1. After dilution with phosphate-buffered saline (PBS) to an appropriate concentration the infection of mice was performed with the indicated dose by i.v. injection into the lateral tail vein.

To estimate bacterial burden of organs, on the indicated time-point after infection mice were sacrificed. Spleens and livers were homogenised in the gentleMACS™ dissociator (Miltenyi Biotec Inc., Bergisch Gladbach, Germany) according to manufacturer’s recommendation. Serial dilution triplicates of Triton-lysed organ homogenates (ranging from 1:10 to 1:1000) were plated on BHI agar plates. After an overnight incubation at 37 °C, colony forming units (CFU) were quantified by counting colonies.

### Sample preparation and flow cytometry

Spleens or thymi were homogenised in the gentleMACS™ dissociator according to manufacturer’s recommendation. Peripheral blood from isoflurane-anesthetised mice was obtained by puncture of the retro-orbital sinus.

Cells were incubated with anti-CD16/32 (2.4G2; BD Biosciences, San Diego, CA, USA) in fluorescence-activated cell sorter (FACS) buffer (PBS, 0.5% bovine serum albumin (BSA), and 0.02% sodium azide, pH 7.45) followed by staining with a combination of fluorescence-conjugated antibodies targeting CD45 (30-F11), CD11b (M1/70), Gr-1 (RB6-8C5), CD19 (1D3), NKp46 (29A1.4), CD3 (17A2), CD4 (RM4-5), CD8 (5H10), CD5 (53–7.3), TCRβ (H57-597), Ep-CAM (G8.8), Ly-51 (6C3), (BioLegend, San Diego, CA, USA; eBioscience, Frankfurt, Germany; BD Biosciences).

MHC multimer reagents for detection of CD8^+^ T cells specific for the immunodominant epitope in BALB/c mice (H-2K^d^-restricted LLO_91–99_ peptide) were generated as described^44^. For live/dead discrimination, cells were incubated with ethidium monazide (EMA; Molecular Probes, Eugene, OR, USA) followed by MHC multimer and surface marker staining.

For intracellular cytokine staining, splenocytes were restimulated for 5 h at 37 °C with LLO_91–99_ (GYKDGNEYI) or with 0.5 μmol/L PMA and 0.25 μg ionomycin, or kept without stimulation (negative control with dimethyl sulfoxide (DMSO), Sigma-Aldrich, Inc., St. Louis, MO, USA) in the presence of Brefeldin A (Golgi Plug, BD Biosciences). Subsequently, cells were incubated with anti-CD16/32, followed by surface staining as described above. After fixation and permeabilization using the Cytofix/Cytoperm kit (BD Biosciences) according to the manufacturer’s recommendation, intracellular staining with anti-IFN-γ (XMG1.2, BD Biosciences) was performed.

Data were acquired on a Gallios flow cytometer (Beckman Coulter Inc., Brea, CA, USA) and further analysed with FLOWJO (Tree Star Inc., Ashland, OR, USA) software.

Flow cytometric analysis of transduced cells was performed as follows: one day after transduction, initial transduction efficacies were assessed by measurement of GFP producing cells. GFP expression was analysed over time as a surrogate marker for MECL-1^WT^ or MECL-1^G170W^ expressing cells. Annexin V staining was performed 36 h after transduction using Pacific Blue™ Annexin V/SYTOX® AADvanced™ Apoptosis Kit (Thermo Scientific, Braunschweig, Germany).

### Analysis of mouse plasma

Plasma samples were stored at −80 °C. Plasma concentrations of immunoglobulins were determined in a bead array-based multiplex assay (Luminex xMAP Technology; Bio-Rad, Munich, Germany), which allows the simultaneous analysis of IgG1, IgG2a, IgG2b, IgG3, IgA, and IgM. The level of each isotype was calculated over a standard curve fitted with Four-Parameter-Logistic regression (Bio-Plex manager software, Bio-Rad).

For determination of TNF-α, IFN-γ, IL-1β, and IL-6 concentrations, the commercial MSD Proinflammatory Panel 1 (mouse) Kit was used according to the manufacturer’s instructions. Data were acquired and analysed using the MSD instrument (Meso QuickPlex SQ120, Meso Scale Discovery, Rockville, MD, USA) and the included MSD discovery workbench software. IFN-α and IFN-β2 plasma concentrations were assessed using 2-Plex Mouse Procarta Panel (Thermo Scientific) according to the manufacturer’s instructions. Data were acquired and analysed on the iQue screener PLUS (Intellicyt, Albuquerque, NM, USA)

### Histology and immunohistochemistry

Immersion-fixed (neutral buffered formalin, 4%) organs were embedded in paraffin using a Vacuum infiltration processor (Sakura TissueTEK model VIP 5E-F2, Tokyo, Japan) and sliced with a HM 335S Rotary Microtome (Thermo Scientific) to a thickness of 4 µm for HE stain or 1 µm for immunohistochemistry. Histological slides were dehydrated in decreasing ethanol series and rinsed in tap water.

For HE staining, slides were incubated for 2 min in Mayers’ acid Hemalum (BioOptica, Milano, Italy), followed by bluing in tap water and immersion in EosinY (BioOptica) for 1 min. Stained slides were dehydrated with increasing ethanol series, mounted with Pertex® (Medite GmbH, Burgdorf, Germany) and coverslipped (Menzel Glaeser®, Braunschweig, Germany).

For immunohistological stainings, antigen retrieval was performed by heat induction (0.1 M EDTA pH 8.0, 25 min at 96 °C/0.1 M citrate pH 6.0 10 min/100 °C under pressure) or Proteinase K incubation (Roche, Basel, Switzerland, diluted 1:200, 8 min, RT). For blocking of unspecific bindings, 5% goat (Gibco, Carlsbad, CA, USA) or rabbit (Gibco) serum was used for 1 h followed by incubation with primary antibodies targeting CD3 (DakoCytomation™, DAKO™, Glostrup, Denmark), B220 (BD Biosciences) or MPO (NeoMarkers, Fremont, CA, USA) at 1:100 dilution overnight at 4 °C. Slides were incubated with biotin conjugated secondary antibodies (α-goat-Biotin, Vector Laboratories, Inc., Burlingame, CA, USA, diluted 1:200 or α-rabbit-Biotin, Vector Laboratories, Inc., diluted 1:750) for 1 h at RT. Detection was performed by incubation with streptavidin-peroxidase (SS HRP Label, BioGenex, Fremont, CA) for 15 min and subsequently 3,3′-diaminobenzidine (DAB, DCS Innovative Diagnostic Systems, Hamburg, Germany) for 3 min. Counterstaining was performed using Mayers’ acid hemalum for 10 sec followed by bluing in tap water. Dehydration and mounting was performed as described above.

### Genetic mapping

Heterozygous TUB6 animals were backcrossed twice with C57BL/6 J mice and the resulting offspring interbred to produce homozygous TUB6 mutants with clearly detectable phenotype. We analysed DNA from 44 phenotypically homozygous TUB6 mutants using 137 single nucleotide polymorphism (SNP) markers^55^. For each marker analysed, the LOD score was determined.

### Capture, exome sequencing, variant calling

We performed in-solution targeted enrichment of exonic sequences from two homozygous TUB6 mice and one wild type littermate control using the SureSelectXT Mouse All Exon 50 Mb kit (Agilent Technologies, Santa Clara, CA, USA). The generated libraries were indexed, pooled and sequenced as 100 bp paired-end runs on a HiSeq2000 system (Illumina, San Diego, CA, USA).

Read alignment to the mouse genome assembly mm9 was done with Burrows-Wheeler Aligner (BWA, version 0.6.1) and yielded 10.6 Gb, 9.1 Gb and 8.3 Gb of mapped sequence data corresponding to an average coverage of 109×, 103× and 76× for the two mutants and the wild type mouse, respectively. Single nucleotide variant (SNV) and small insertion and deletions (indel) detection with SAMtools (version 0.1.18) yielded 14,278, 14,256 and 14,254 good quality non-synonymous coding variants. After filtering for homozygous variants present in the two TUB6 mice but not in the wild type mouse and 145 control mice with unrelated phenotypes, a single missense variant in the gene *Psmb10* remained.

### Cloning of proteasome mutants

The *PUP1-TEV-ProA-His*_*7*_ cassette^56^ including 503 bps of the endogenous *PUP1* promoter and 229 bps of the tADH1 terminator was amplified from genomic DNA isolated from the yeast strain W303-*PUP1-TEV-ProA-His*_*7*_ (M. Groll, unpublished results) using the primers Pup1_for and Pup1_rev. The *Xba*I and *Hin*dIII digested PCR (polymerase chain reaction) product was inserted into the vector pRS315, carrying the *LEU2* selection marker, to create pRS315-*PUP1-TEV-ProA-His*_*7*_. Point mutants were created by QuikChange site-directed mutagenesis using either the plasmid pRS315-*PUP1*^40^ or pRS315-*PUP1-TEV-ProA-His*_*7*_ as template DNA. Introduction of point mutations was confirmed by sequencing. The mutant pRS315-*pup1-TEV-ProA-His*_*7*_ and pRS315-*pup1* plasmids were introduced into the yeast strain YWH10 *pup1Δ*::*HIS3* [pRS316-*PUP1*]^41^, which is chromosomally deleted for the *PUP1* gene and instead carries a *URA3-PUP1*-episome. After growth on synthetic complete medium without leucine (CM leu^−^) transformants were selected on 5-fluoroorotic acid (5-FOA) for loss of the wild type *URA3-PUP1*-plasmid. Hereby, only yeasts encoding a mutant but functional *pup1* gene can survive. Oligonucleotides, plasmids and yeast strains are listed in the Tables S2–S4.

### Yeast drop test

Yeast strains were grown over night at 30 °C in liquid yeast extract peptone dextrose (YPD) medium. Serial dilutions of cells were spotted on YPD plates. Growth of the strains was evaluated after 1 and 3 days at 30 °C and 37 °C.

### Proteasome purification

Mutant yeast strains were grown in 18 l cultures at 30 °C in YPD into early stationary phase. Cells were resuspended in 100 mM Tris/HCl pH 7.5, 500 mM NaCl, 20 mM imidazole and disrupted by French Press. Cellular debris was pelleted by centrifugation for 30 min at 41,000 g and the cleared cellular extract was loaded onto a Ni^2+^-NTA chromatography column. The proteasome was eluted by applying a linear gradient to 500 mM imidazole. The eluted protein fractions were immediately diluted in 100 mM Tris/HCl pH 7.5 and 20% (v/v) glycerol in a 1:1 ratio, dialyzed against 20 mM Tris/HCl pH 7.5 and 50 mM NaCl and loaded onto an anion exchange chromatography column. To identify proteasome-containing fractions, protein samples were tested for their proteolytic activity against the fluorogenic substrate Suc-LLVY-AMC (*N*-Succinyl-Leu-Leu-Val-Tyr-7-Amino-4-methylcoumarin). Active fractions were pooled and subjected to size exclusion chromatography (Superose 6 10/300; 20 mM Tris/HCl pH 7.5, 100 mM NaCl). After incubation with TEV protease in a molar ratio of 1:100 for 15 h at 4 °C to remove the ProteinA-H_7_-tag, the samples were concentrated with a 100 kDa cut-off filter. This procedure separated the mutant proteasome from the TEV protease and the cleaved tag and allowed buffer exchange to 10 mM 2-(*N*-morpholino)ethanesulfonic acid (MES) pH 6.8.

### Crystallisation and structure determination

Crystals were grown by the hanging drop vapour diffusion technique at 20 °C. Protein (33 mg/ml) and reservoir solutions (100 mM MES pH 6.8, 20 mM magnesium acetate pH 6.8, 13 (v/v)% 2-methyl-2,4-pentanediol (MPD)) were mixed in a 2:1 ratio. Crystals were cryoprotected with 5 µl 100 mM MES pH 6.8, 20 mM magnesium acetate pH 6.8, 30% (v/v) MDP and either vitrified in liquid nitrogen immediately or after soaking with bortezomib for 24 h in a final concentration of 1.5 mM.

Diffraction data were collected using synchrotron radiation of λ = 1.0 Å at the beamline X06SA, Swiss Light Source (SLS), Villigen, Switzerland. X-ray intensities were analysed with the program package XDS^57^. The yeast wild type proteasome structure (PDB ID 5CZ4)^58^ served as a search model for structure determination by molecular replacement with PHASER^59^. Cyclic refinement and model building were conducted with REFMAC5^60^ and COOT^61^. TLS (translation/libration/screw) refinements with restraints between bonded and non-crystallographic symmetry-related atoms finally yielded excellent values for R_crys_, R_free_, r.m.s.d. bond and angle values as well as good stereochemistry from the Ramachandran Plot (Table S1).

### Overexpression experiments

For overexpression experiments in yeast, *pup1* gene versions were cloned from the pRS315 plasmids into the *LEU2*-marked high copy 2 µ plasmid pRS425^62^ using the restriction endonucleases *Hin*dIII and *Sac*I. Correct plasmids were transformed into the wild type yeast strain WCG4a and transformants were selected on synthetic CM leu^−^ plates.

For overexpression experiments in murine cells, spleen mRNA from a heterozygous TUB6 mouse was isolated using TriReagent (Sigma-Aldrich) and transcribed to cDNA using Affinity Script Multiple Temperature cDNA synthesis kit (Agilent, Santa Clara, CA, USA). MECL-1^WT^ and MECL-1^G170W^ cDNA was amplified and linked to P2A GFP using an overlap extension method. MECL-1^WT^-P2A-GFP and MECL-1^G170W^-P2A-GFP were subsequently cloned into the mP71 retroviral expression vector. Platinum E cells cultured in DMEM medium (Thermo Scientific) supplemented with 10% FCS (fetal calf serum), 0.025% L-glutamine, 0.1% HEPES (4-(2-hydroxyethyl)-1-piperazineethanesulfonic acid), 0.001% gentamycin and 0.002% streptomycin were transiently transfected with the MECL-1^WT^-P2A-GFP and MECL-1^G170W^-P2A-GFP constructs using a calcium phosphate precipitation based method. Virus supernatant was harvested after 48 h. Subsequently a second virus supernatant was produced for 24 h in RPMI 1640 medium (Thermo Scientific) supplemented with 10% FCS, 0.025% L-glutamine, 0.1% HEPES, 0.001% gentamycin and 0.002% streptomycin. 24-well plates were coated with retronectin (6.25 µg/ml, Takara Bio Europe S.A.S., Saint-Germain-en-Laye, France), anti-CD3e (0.5 µg/ml, BD Biosciences, Clone 145.2C11) and anti-CD28 (0.1 µg/ml, BD Biosciences, Clone 37.51) diluted in PBS at 4 °C overnight. Splenocytes were cultured in supplemented RPMI 1640 containing anti-CD3e (0.5 µg/ml), anti-CD28 (0.1 µg/ml) and human IL-2 (20 U/ml, Proleukin® S, Novartis, Basel, Switzerland) for 24 h prior to transduction. On the day of transduction, diluted retronectin was removed and plates were coated with DMEM virus supernatant by centrifugation with 3000 g at 4 °C. DMEM virus supernatant was removed and splenocytes were resuspended in RPMI virus supernatant supplemented with human IL-2 (25 U/ml). Spinoculation was performed at 800 g, 32 °C for 1.5 h. Transduced T cells were subsequently cultured in supplemented RPMI 1640 with 25 ng/ml human IL-15 (PeproTech Germany, Hamburg, Germany) and 25 U/ml human IL-2 or left in the anti-CD3e, anti-CD28 coated plates replacing the medium every two days with fresh medium containing 25 U/ml human IL-2.

### Yeast cell lysate preparation

Yeast strains were grown in YPD medium at 30 °C and harvested during log-phase growth (OD_600_ 1–2).

Native yeast cell lysates were prepared by resuspending 3·10^8^ cells in 100 µl lysis buffer (50 mM Tris/HCl pH 8.0, 5 mM MgCl_2_, 0.5 mM EDTA, pH 8.0, 1 mM ATP, AEBSF)^62^. Upon vortexing with acid washed glass beads (Sigma-Aldrich, 0.5 mm) for 5 × 1 min (with incubations on ice in between), the lysate was cleared by centrifugation (30 min, 15,000 g, 4 °C). The resulting supernatant was used for native gel analysis.

Denatured yeast protein extracts were prepared by dissolving 10^8^ cells in 80 µl SDS-loading buffer (freshly supplemented with β-mercaptoethanol). The cells were shortly vortexed, boiled for 20 min at 95 °C and afterwards subjected to SDS-PAGE analysis.

### Polyacrylamide gel electrophoresis and immunoblotting of yeast lysates

Native yeast lysates were mixed in a 1:1 ratio with native sample buffer (62.5 mM Tris-HCl pH 6.8, 40% (v/v) glycerol, 0.01% (w/v) bromophenol blue) and loaded on 4–15% gradient native gels (Mini-PROTEAN TGX Precast Gels, Biorad). Native gels were run for 3 h at 25 mA and 4 °C in native electrophoresis buffer (25 mM Tris, 192 mM glycine).

15% SDS gels were run for 40 min at 25 mA and 20 °C in standard electrophoresis buffer (25 mM Tris, 192 mM glycine, 0.1% (w/v) SDS). To distinguish between mature and immature yβ2 subunits, gels were run up to 2 h until the marker bands between 32 and 80 kDa (NEB, Color Prestained Protein Standard Broad Range) were properly resolved.

Prior to blotting, native gels were incubated for 20 min in transfer buffer^63^ containing 2.4% (w/v) SDS. Semi-dry blotting of native gels onto nitrocellulose membranes was performed for 3 h at 80 mA and 20 °C using transfer buffer. Afterwards, the membrane was incubated in 25 mM NaOH for 30 min and boiled for a few minutes. Blocking was performed for 1 h at 20 °C with 5% (w/v) milk powder dissolved in TBS (Tris-buffered saline) supplemented with 0.5% (v/v) Tween20 (T). Anti-β7 serum^41^ was applied in a 1:2,500 dilution in TBS-T (0.5% (v/v)) for at least 1 h at 20 °C. An anti-rabbit horseradish peroxidase (HRP) conjugate (A16110, Thermo Scientific) served as secondary antibody (1:10,000 dilution in 5% (w/v) milk TBS-T (0.1% (v/v))).

SDS gels were transferred onto nitrocellulose membranes for 1 h at 100 mA. The membrane was blocked for 1 h at 20 °C with 5% (w/v) BSA dissolved in TBS supplemented with 0.1% (v/v) Tween20. The anti-His antibody (#2365, Cell signalling) was diluted 1:3,000 in 5% (w/v) BSA in TBS-T (0.1% (v/v)) and applied overnight at 4 °C. The secondary antibody, an anti-rabbit HRP conjugate (A16110, Thermo Scientific) was used in a 1:10,000 dilution in 5% (w/v) BSA TBS-T (0.1% (v/v)).

Upon enhanced chemiluminescence (ECL) development using Western Bright ECL-Spray (Advansta, Menlo Park, CA, USA) chemoluminescence was recorded by the ImageQuant LAS4000 biomolecular imager (GE Healthcare, Chalfont St Giles, UK).

Cleavage of the ProA-H_7_ tag by TEV protease was proven by anti-ProA western blotting. Upon blocking with 5% (w/v) milk powder in TBS-T (0.1% (v/v)), anti-Protein A antibody (ABIN223591, Antikörper-online) was added to the blocking solution in a 1:2,000 dilution. The secondary antibody, an anti-rabbit alkaline phosphatase conjugate (A3687, Sigma-Aldrich) was applied in a 1:10,000 dilution in 5% (w/v) milk powder-TBS-T (0.1% (v/v)). Upon washing with TBS, colorimetric detection was started by the addition of NBT (Nitro blue tetrazolium chloride; 0.3 mg/ml) and 5-Bromo-4-chloro-3-indolyl phosphate (BCIP; 0.16 mg/ml) in buffer containing 100 mM Tris/HCl pH 9.6, 100 mM NaCl and 5 mM MgCl_2_.

### SDS-PAGE and western blot of murine organs

Mouse organs were lysed (10 mM Tris HCl pH 7.8, 150 mM NaCl, 1% Triton X-100 and Complete Protease Inhibitor Cocktail (Roche)) on ice for 20 min. Lysates were centrifuged at 20,000 g and 4 °C for 15 min. Supernatants were mixed with SDS sample buffer and boiled for 5 min at 95 °C. Proteins were separated by SDS-PAGE (15% gel) and blotted onto nitrocellulose membrane (Whatman). Membranes were blocked for 1 h (5% milk powder/0.2% Tween20) followed by overnight incubation in primary antibodies (α-MECL-1^64^ or α-iota (clone IB5, recognises the α1-subunit iota of the proteasome and was obtained from Prof. Dr. Klaus Scherrer (Institut JacquesMonod, Paris, France))) at 4 °C. Membranes were washed and incubated for 2 h with appropriate peroxidase-conjugated secondary antibodies (Dako). Membranes were washed and proteins were visualised with ECL.

## Electronic supplementary material

Supplementary Information

## References

[1] Hershko A, Ciechanover A, Varshavsky A (2000). Basic medical research award. The ubiquitin system. Nat. Med..

[2] Pamer E, Cresswell P (1998). Mechanisms of MHC class I-restricted antigen processing. Annu. Rev. Immunol..

[3] Huber EM (2012). Immuno- and constitutive proteasome crystal structures reveal differences in substrate and inhibitor specificity. Cell.

[4] Marques AJ, Palanimurugan R, Matias AC, Ramos PC, Dohmen RJ (2009). Catalytic mechanism and assembly of the proteasome. Chem. Rev..

[5] Groettrup M, Kirk CJ, Basler M (2010). Proteasomes in immune cells: more than peptide producers?. Nat. Rev. Immunol..

[6] Nandi D, Jiang H, Monaco JJ (1996). Identification of MECL-1 (LMP-10) as the third IFN-gamma-inducible proteasome subunit. J. Immunol..

[7] Aki M (1994). Interferon-gamma induces different subunit organizations and functional diversity of proteasomes. J. Biochem..

[8] Basler M, Moebius J, Elenich L, Groettrup M, Monaco JJ (2006). An altered T cell repertoire in MECL-1-deficient mice. J. Immunol..

[9] Fehling HJ (1994). MHC class I expression in mice lacking the proteasome subunit LMP-7. Science.

[10] Van Kaer L (1994). Altered peptidase and viral-specific T cell response in LMP2 mutant mice. Immunity.

[11] Kincaid EZ (2012). Mice completely lacking immunoproteasomes show major changes in antigen presentation. Nat. Immunol..

[12] Murata S (2007). Regulation of CD8+ T cell development by thymus-specific proteasomes. Science.

[13] Xing Y, Jameson SC, Hogquist KA (2013). Thymoproteasome subunit-beta5T generates peptide-MHC complexes specialized for positive selection. Proc. Natl. Acad. Sci. USA.

[14] De M (2003). Beta 2 subunit propeptides influence cooperative proteasome assembly. J. Biol. Chem..

[15] Guillaume B (2010). Two abundant proteasome subtypes that uniquely process some antigens presented by HLA class Imolecules. Proc. Natl. Acad. Sci. USA.

[16] de Bruin G, Xin BT, Florea BI, Overkleeft HS (2016). Proteasome subunit selective activity-based probes report on proteasome core particle composition in a native polyacrylamide gel electrophoresis fluorescence-resonance energy transfer assay. J. Am. Chem. Soc..

[17] Gomes AV (2013). Genetics of proteasome diseases. Scientifica.

[18] McDermott A, Jacks J, Kessler M, Emanuel PD, Gao L (2015). Proteasome-associated autoinflammatory syndromes: advances in pathogeneses, clinical presentations, diagnosis, and management. Int. J. Dermatol..

[19] Kitamura A (2011). A mutation in the immunoproteasome subunit PSMB8 causes autoinflammation and lipodystrophy in humans. J. Clin. Invest..

[20] Liu Y (2012). Mutations in proteasome subunit beta type 8 cause chronic atypical neutrophilic dermatosis with lipodystrophy and elevated temperature with evidence of genetic and phenotypic heterogeneity. Arthritis Rheum..

[21] Garg A (2010). An autosomal recessive syndrome of joint contractures, muscular atrophy, microcytic anemia, and panniculitis-associated lipodystrophy. J. Clin. Endocrinol. Metab..

[22] Arima K (2011). Proteasome assembly defect due to a proteasome subunit beta type 8 (PSMB8) mutation causes the autoinflammatory disorder, Nakajo-Nishimura syndrome. Proc. Natl. Acad. Sci. USA.

[23] Brehm A (2016). Additive loss-of-function proteasome subunit mutations in CANDLE/PRAAS patients promote type I IFN production. J. Clin. Invest..

[24] Kunjappu MJ, Hochstrasser M (2014). Assembly of the 20S proteasome. Biochim. Biophys. Acta.

[25] Gallastegui N, Groll M (2010). The 26S proteasome: assembly and function of a destructive machine. Trends Biochem. Sci..

[26] Murata S, Yashiroda H, Tanaka K (2009). Molecular mechanisms of proteasome assembly. Nat. Rev. Mol. Cell Biol..

[27] Ditzel L (1998). Conformational constraints for protein self-cleavage in the proteasome. J. Mol. Biol..

[28] Hirano Y (2008). Dissecting beta-ring assembly pathway of the mammalian 20S proteasome. EMBO J.

[29] Ramos PC, Marques AJ, London MK, Dohmen RJ (2004). Role of C-terminal extensions of subunits beta2 and beta7 in assembly and activity of eukaryotic proteasomes. J. Biol. Chem..

[30] Kloetzel PM (2004). Generation of major histocompatibility complex class I antigens: functional interplay between proteasomes and TPPII. Nat. Immunol..

[31] Hrabe de Angelis MH (2000). Genome-wide, large-scale production of mutant mice by ENU mutagenesis. Nat. Genet..

[32] Beutler B (2006). Genetic analysis of host resistance: Toll-like receptor signaling and immunity at large. Annu. Rev. Immunol..

[33] Barbaric I, Wells S, Russ A, Dear TN (2007). Spectrum of ENU-induced mutations in phenotype-driven and gene-driven screens in the mouse. Environ. Mol. Mutagen..

[34] Gailus-Durner V (2005). Introducing the German Mouse Clinic: open access platform for standardized phenotyping. Nat. Methods.

[35] Fuchs H (2012). Innovations in phenotyping of mouse models in the German Mouse Clinic. Mamm. Genome.

[36] Flaswinkel H (2000). Identification of immunological relevant phenotypes in ENU mutagenized mice. Mamm. Genome.

[37] Sun M (2012). Multiplex Chromosomal Exome Sequencing Accelerates Identification of ENU-Induced Mutations in the Mouse..

[38] Fairfield H (2011). Mutation discovery in mice by whole exome sequencing. Genome Biol..

[39] Groll M (1997). Structure of 20S proteasome from yeast at 2.4 A resolution. Nature.

[40] Groll M (1999). The catalytic sites of 20S proteasomes and their role in subunit maturation: a mutational and crystallographic study. Proc. Natl. Acad. Sci. USA.

[41] Heinemeyer W, Fischer M, Krimmer T, Stachon U, Wolf DH (1997). The active sites of the eukaryotic 20 S proteasome and their involvement in subunit precursor processing. J. Biol. Chem..

[42] Pamer EG (2004). Immune responses to Listeria monocytogenes. Nature reviews. Immunology.

[43] Bancroft GJ, Schreiber RD, Bosma GC, Bosma MJ, Unanue ER (1987). A T cell-independent mechanism of macrophage activation by interferon-gamma. J. Immunol..

[44] Busch DH, Pilip IM, Vijh S, Pamer EG (1998). Coordinate regulation of complex T cell populations responding to bacterial infection. Immunity.

[45] Rode I, Boehm T (2012). Regenerative capacity of adult cortical thymic epithelial cells. Proc. Natl. Acad. Sci. USA.

[46] Manz MG, Boettcher S (2014). Emergency granulopoiesis. Nat. Rev. Immunol..

[47] Murata S, Takahama Y, Tanaka K (2008). Thymoproteasome: probable role in generating positively selecting peptides. Curr. Opin. Immunol..

[48] Bai M (2014). Assembly mechanisms of specialized core particles of the proteasome. Biomolecules.

[49] Chen GY, Nunez G (2010). Sterile inflammation: sensing and reacting to damage. Nat. Rev. Immunol..

[50] Nitta T (2015). The thymic cortical epithelium determines the TCR repertoire of IL-17-producing gammadeltaT cells. EMBO Rep..

[51] Cook MC, Vinuesa CG, Goodnow CC (2006). ENU-mutagenesis: insight into immune function and pathology. Curr. Opin. Immunol..

[52] Soewarto D, Klaften M, Rubio-Aliaga I (2009). Features and strategies of ENU mouse mutagenesis. Curr. Pharm. Biotechnol..

[53] Rathkolb B (2000). Large-scale N-ethyl-N-nitrosourea mutagenesis of mice-from phenotypes to genes. Exp. Physiol..

[54] Slack E (2009). Innate and adaptive immunity cooperate flexibly to maintain host-microbiota mutualism. Science.

[55] Klaften M, Hrabe de Angelis M (2005). ARTS: a web-based tool for the set-up of high-throughput genome-wide mapping panels for the SNP genotyping of mouse mutants. Nucleic Acids Res..

[56] Knop, M. *et al*. Epitope tagging of yeast genes using a PCR-based strategy: more tags and improved practical routines. *Yeast* **15**, 963–972, 10.1002/(SICI)1097-0061(199907)15:10B 963::AID-YEA399 3.0.CO;2-W (1999). 10.1002/(SICI)1097-0061(199907)15:10B<963::AID-YEA399>3.0.CO;2-W 10407276

[57] Kabsch W (2010). XDS. Acta Crystallogr. D.

[58] Huber EM (2016). A unified mechanism for proteolysis and autocatalytic activation in the 20S proteasome. Nat. Commun..

[59] McCoy AJ (2007). Phaser crystallographic software. J. Appl. Cryst..

[60] Vagin AA (2004). REFMAC5 dictionary: organization of prior chemical knowledge and guidelines for its use. Acta Crystallogr. D.

[61] Emsley P, Lohkamp B, Scott WG, Cowtan K (2010). Features and development of Coot. Acta Crystallogr. D.

[62] Christianson TW, Sikorski RS, Dante M, Shero JH, Hieter P (1992). Multifunctional yeast high-copy-number shuttle vectors. Gene.

[63] Elsasser S, Schmidt M, Finley D (2005). Characterization of the proteasome using native gel electrophoresis. Methods Enzymol..

[64] Groettrup M, Standera S, Stohwasser R, Kloetzel PM (1997). The subunits MECL-1 and LMP2 are mutually required for incorporation into the 20S proteasome. Proc. Natl. Acad. Sci. USA.

