# Supplementary material for: Defective immuno- and thymoproteasome assembly causes severe immunodeficiency
Source: Sci Rep. 2018 Apr 13;8:5975. doi: 10.1038/s41598-018-24199-0 (PMC5899138; doi:10.1038/s41598-018-24199-0)
Supplement: Supplementary file 1 — Supplementary Information [file 41598_2018_24199_MOESM1_ESM.pdf]

Defective immuno- and thymoproteasome assembly causes severe immunodeficiency

Irina Treise, Eva M. Huber, Tanja Klein-Rodewald, Wolfgang Heinemeyer, Simon A. Grassmann, Michael Basler, Thure Adler, Birgit Rathkolb, Laura Helming, Christian Andres, Matthias Klaften, Christina Landbrecht, Thomas Wieland, Tim M. Strom, Kathy D. McCoy, Andrew J. Macpherson, Eckhard Wolf, Marcus Groettrup, Markus Ollert, Frauke Neff, Valerie Gailus-Durner, Helmut Fuchs, Martin Hrabě de Angelis, Michael Groll, Dirk H. Busch

Supplementary Information

Content

Supplementary Figures S1-S9
and Supplementary Figure Legends
Supplementary Tables S1-S4
Supplementary Methods
Additional References

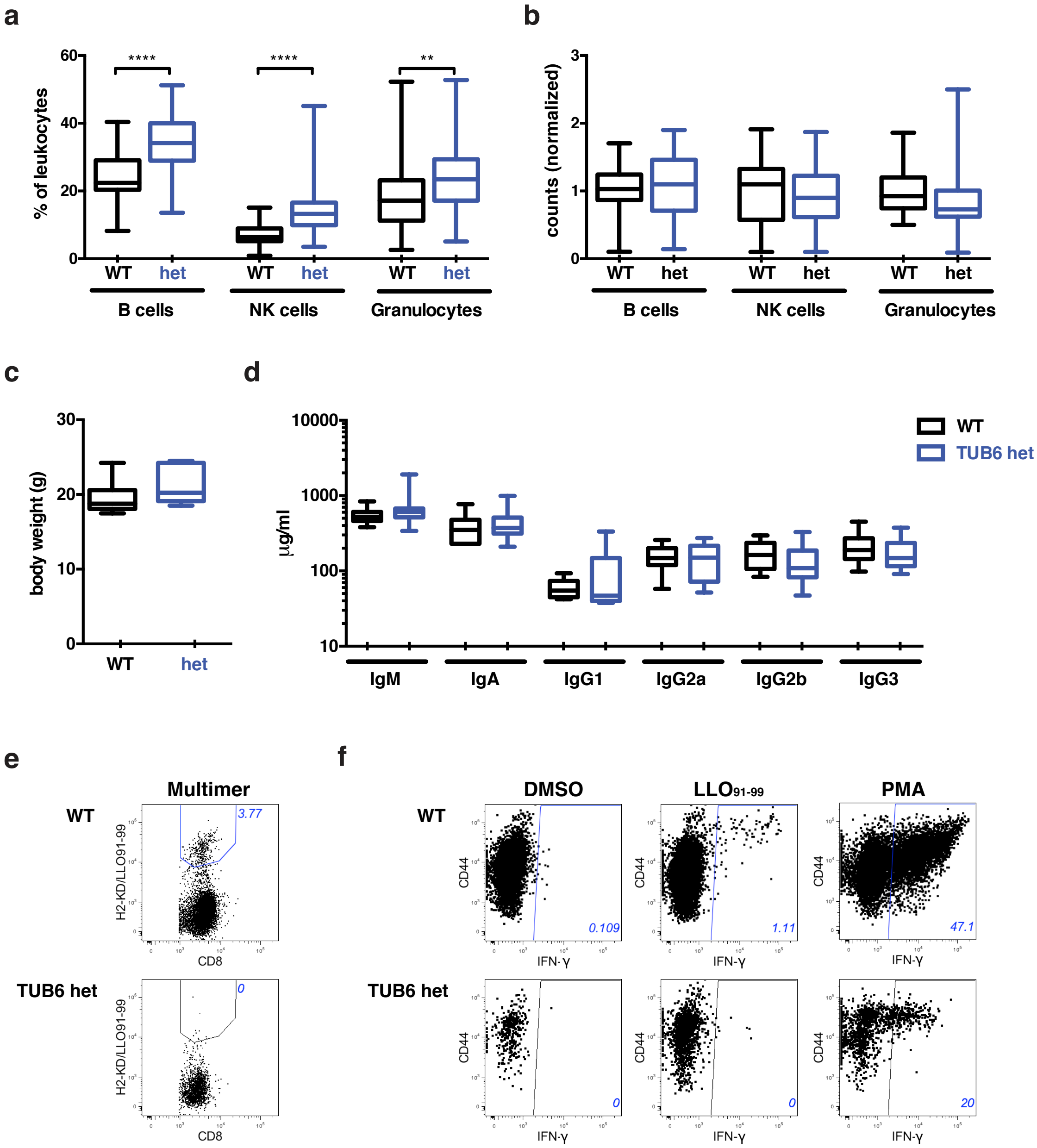

Supplementary Figure S1: Phenotype of heterozygous TUB6 mutants characterised by numerical and functional T cell defect

a,b) Leukocyte frequencies (a) and numbers (b) from peripheral blood of heterozygous TUB6 mutants and wild type controls. B cells ($CD19^+B220^+$), NK cells ($NKp46^+$) and granulocytes ($CD11b^+Gr\text{-}1^+$). Cell counts are normalised to the mean of the wild type group (b). c) Body weight of 6-8 weeks old male heterozygous TUB6 mutants and wild type littermates. d) Plasma immunoglobulin titers of 12-week old mice. e,f) T cell analysis on day 7 upon *L.m.* infection as antigen-specific $CD8^+$ T cells identified by $H\text{-}2K^d/LLO_{91\text{-}99}$ multimer staining (e) and IFN-γ producing $CD8^+$ T cells upon stimulation with DMSO (negative control), peptide $LLO_{91\text{-}99}$ or PMA and ionomycin (f). Plots are gated on living $CD45^+$ $CD3^+$ $CD8^+$ cells; for heterozygotes, merged data from 5 individual mice are shown.

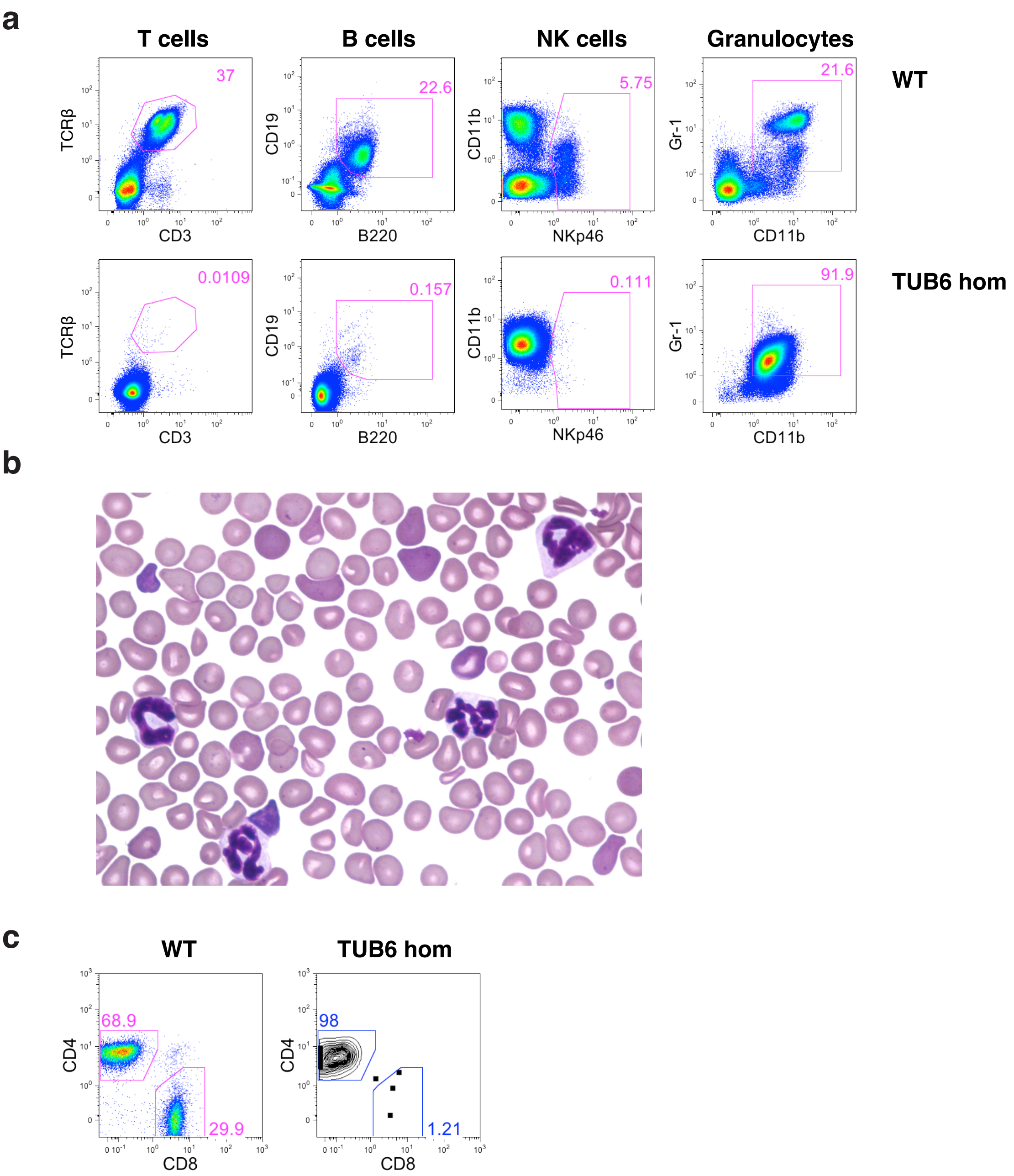

Supplementary Figure S2: Phenotypic characterisation of homozygous TUB6 mutants

a) Representative flow cytometric profile of peripheral blood leukocytes. b) Microscopic analysis of blood smears from representative homozygous TUB6 mouse showing neutrophil granulocytes. c) $CD4^{+}$ vs. $CD8^{+}$ profile of residual T cells shows strong shift towards CD4 in blood samples from homozygous TUB6 mutants. Plots are pregated on $CD45^{+}$ $CD3^{+}$ $TCR\beta^{+}$ cells.

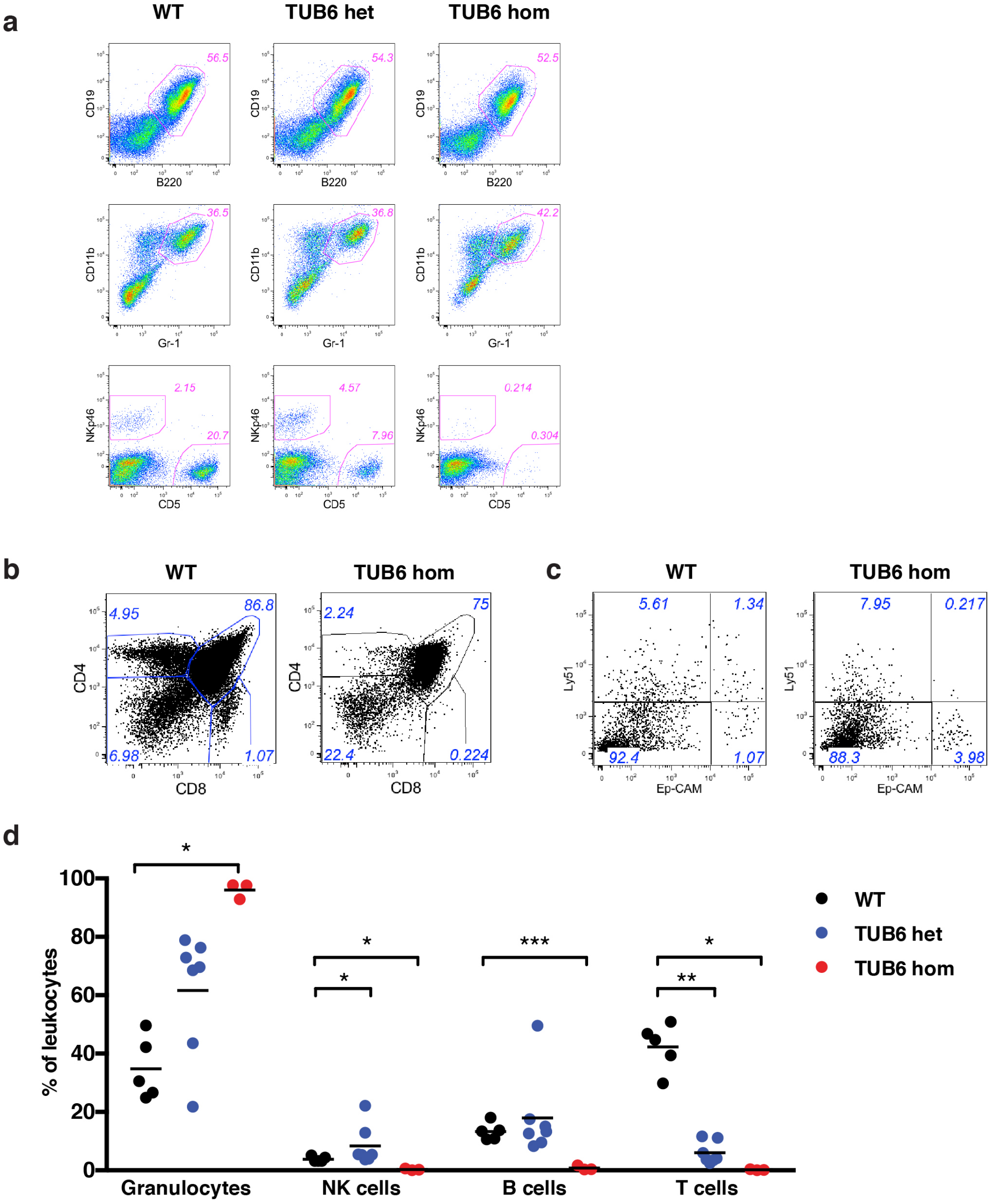

Supplementary Figure S3: Phenotype of neonatal TUB6 mutants and persistence of the TUB6 phenotype under germ-free breeding conditions

a-c) Flow cytometric analysis of indicated cell populations of 2-5 days old pups: splenic leukocyte populations (a), thymocytes (b) and thymic epithelial cells (TECs; c) shown as representative dot plots. d) Germ-free TUB6 mutants represent a phenocopy of mutants with a normal commensal flora. Flow cytometric evaluations of peripheral blood leukocytes are shown.

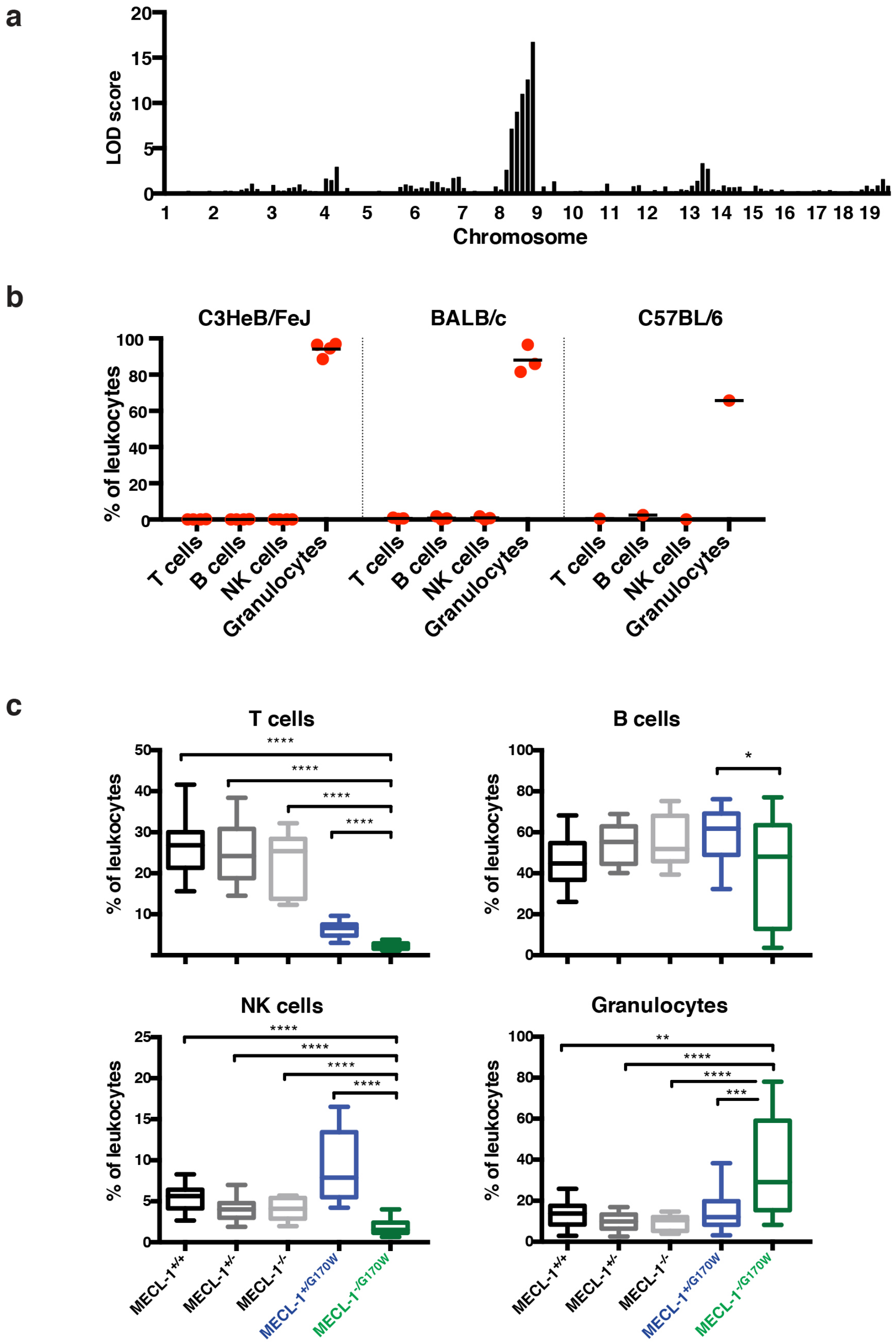

Supplementary Figure S4: Identification and validation of the underlying MECL-1 mutation

a) Genetic mapping of the TUB6 mutation by single nucleotide polymorphism (SNP) analysis between C57BL/6J and C3H/HeJ strains. b) Phenotype persistence after backcross to BALB/c (F11) and C57BL/6 (F7). c) Validation of the mutation by phenotyping of the TUB6 x MECL-1 KO offspring. Flow cytometric evaluation of leukocyte frequencies in peripheral blood of wild type (MECL-$1^{+/+}$), heterozygous and homozygous MECL-1 knockout (MECL-$1^{+/-}$ and MECL-$1^{-/-}$), heterozygous TUB6 (MECL-$1^{+/G170W}$), and hemizygous (MECL-$1^{-/G170W}$) mice.

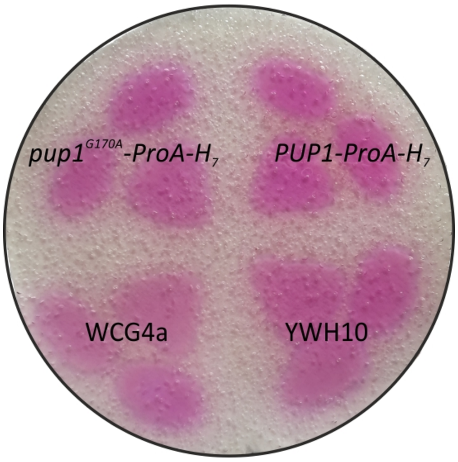

Supplementary Figure S5: Analysis of proteasome activity *in situ*

Test for the chymotrypsin-like (ChT-L; β5) activity of WT and mutant yCP. Yeast strains WCG4a, YWH10 and YEH38 (*pup1Δ::HIS3* [pRS315-*PUP1-ProA-*H_7]), expressing WT yβ2, served as positive controls. Intense pink colouring is indicative of WT-like ChT-L activity towards the substrate Z-GGL-pNA. ChT-L activity correlates with the amount of matured CPs, as CP subunits that are not part of functional CPs are proteolytically inactive.

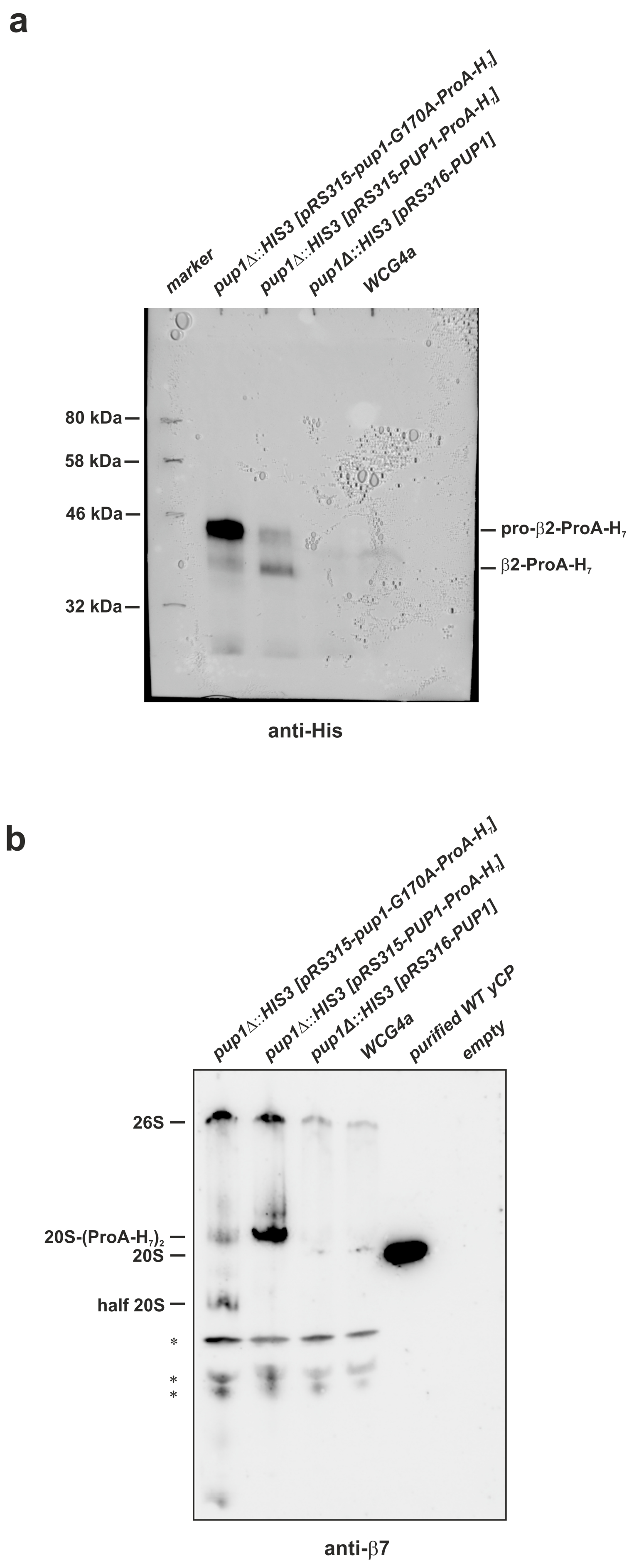

a
marker
pup1Δ::HIS3 [pRS315-pup1-G170A-ProA-H7]
pup1Δ::HIS3 [pRS315-PUP1-ProA-H7]
pup1Δ::HIS3 [pRS316-PUP1]
WCG4a
80 kDa
58 kDa
46 kDa
32 kDa
pro-β2-ProA-H7
β2-ProA-H7
anti-His
b
pup1Δ::HIS3 [pRS315-pup1-G170A-ProA-H7]
pup1Δ::HIS3 [pRS315-PUP1-ProA-H7]
pup1Δ::HIS3 [pRS316-PUP1]
WCG4a
purified WT yCP
empty
26S
20S-(ProA-H7)2
20S
half 20S
*
*
*
anti-β7

Supplementary Figure S6: Full-length versions of western blots shown in Figure 6

a) Anti-His immunoblotting of β2 (*pup1*) G170A-ProA-H_7 and β2 (*PUP1*)-ProA-H_7 cell lysates separated by SDS-PAGE. WCG4a and *pup1Δ::HIS3* [pRS316-*PUP1*] cell lysates that express untagged β2 subunits served as negative controls. Molecular size markers are indicated. b) Anti-β7 western blot of cell lysates separated by native PAGE. As a positive control purified WT yeast 20S proteasome (yCP) was used. 20S proteasomes from β2-ProA-His_7 tagged strains migrate slower than native 20S particles due to their increased size. ProA-His_7 tagged yeasts generally express higher levels of 20S proteasomes than WCG4a and *pup1Δ::HIS3* [pRS316-*PUP1*] cells. In the latter strains proteasomes are mostly present as 26S version. Bands arising from non-specific binding of the antibody are marked by asterisks. Note that there is no marker for native PAGE analysis.

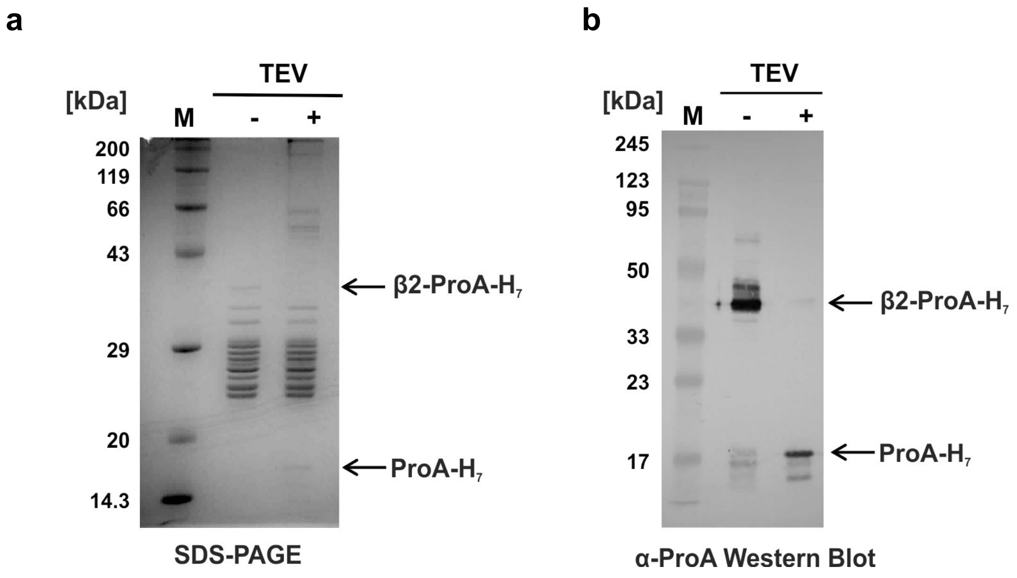

Supplementary Figure S7: Proteasome purification

a) SDS-PAGE of purified β2-G170A-ProA-His_7 mutant yCP prior and after the addition of TEV protease. b) Anti-ProA western blotting confirms the removal of the C-terminal ProA-His_7-tag from subunit yβ2.

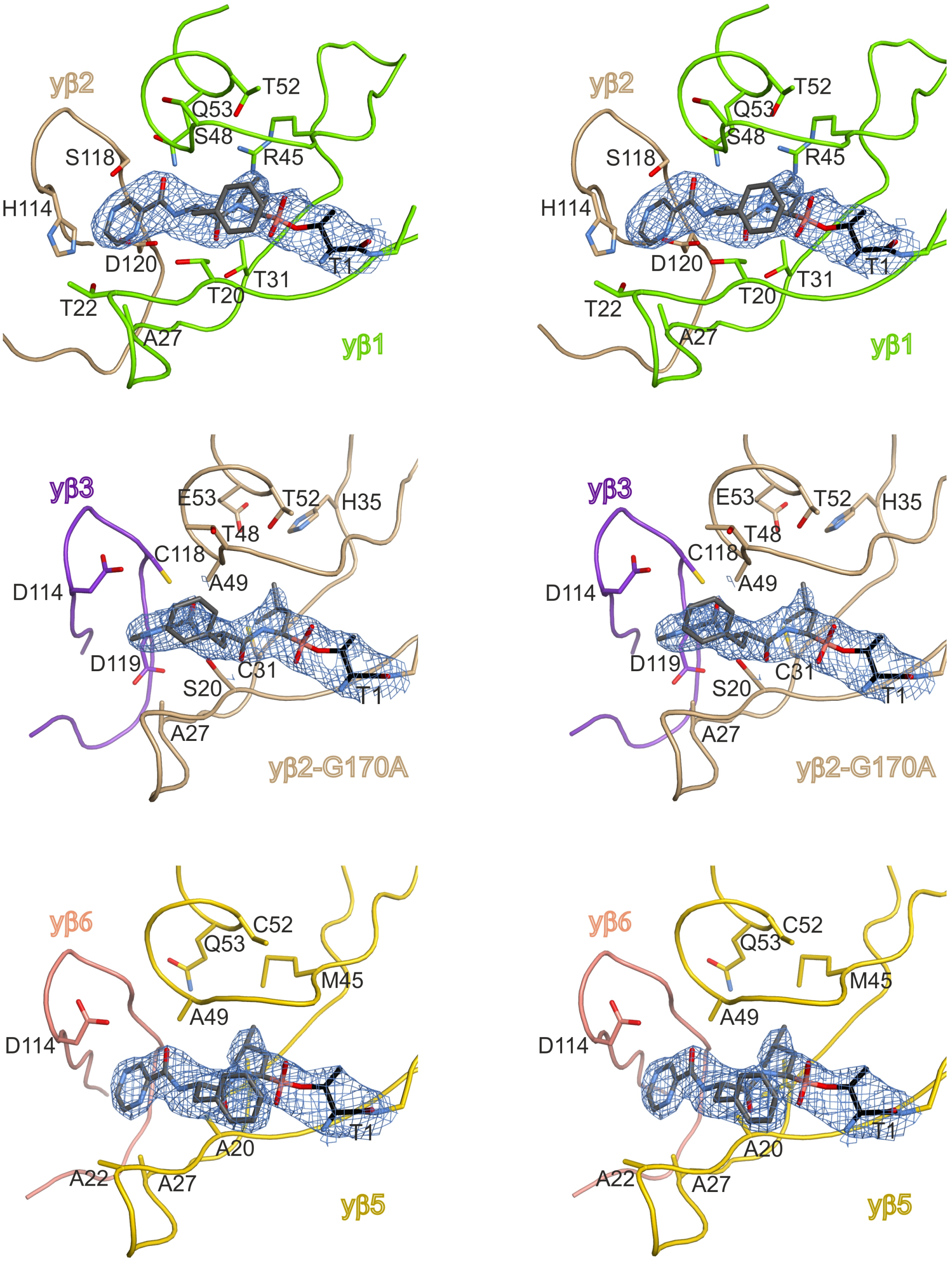

Supplementary Figure S8: X-ray structure of the β2-G170A mutant yCP

Stereo representation of the $2F_O$-F_C electron density maps (contoured at 1σ) for the inhibitor bortezomib covalently bound to the active site Thr1 of the proteasomal subunits yβ1, yβ2-G170A, yβ5.

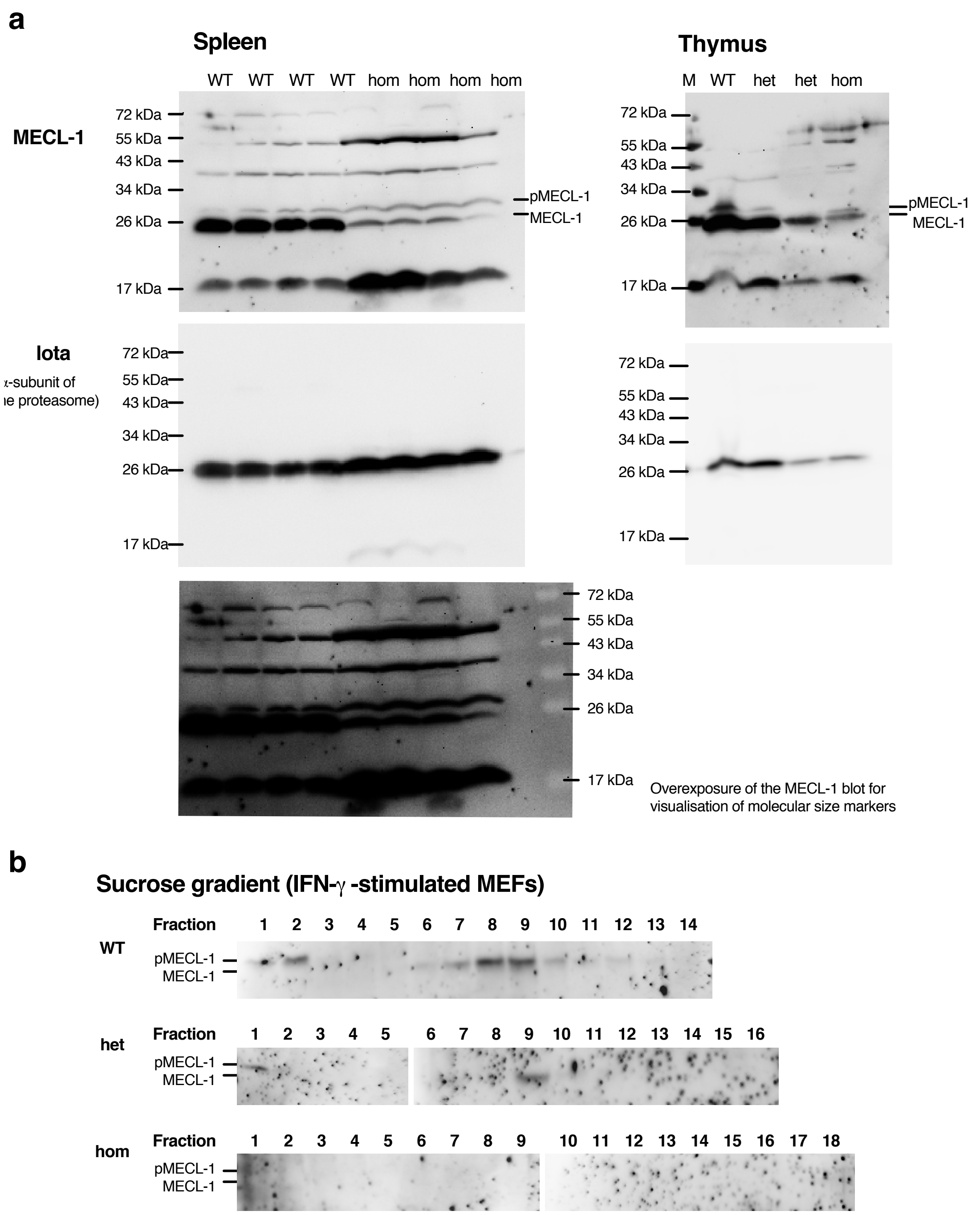

Supplementary Figure S9: MECL-1 expression in homozygous TUB6 tissues

a) Anti-MECL-1 western blotting of spleen and thymus lysates from 3-4-weeks-old mice (BALB/c background), proteasome subunit iota (α1) was used as loading control. The upper pMECL-1 band indicates the immature MECL-1 proprotein containing its propeptide. The lower MECL-1 band indicates the fully processed MECL-1 after assembly of the half-CPs and cleavage of the propeptide.

b) Anti-MECL-1 western blotting of IFN-γ stimulated (24 h) MEF lysates separated on sucrose gradients. In WT and heterozygous TUB6 MEFs, the monomeric MECL-1 precursors are visible in fractions 1 and 2 and mature processed MECL-1 is found in factions 8 and 9. In heterozygous MEFs, the mature processed MECL-1 found in factions 8 and 9 is reduced compared to WT MEFs and an accumulation of the monomeric MECL-1 precursors can be observed in fraction 1. In gradients performed with MEFs from homozygous TUB6 mice no signal was obtained for MECL-1.

Supplementary Tables

Table S1: X-ray data collection and refinement statistics

	yCP β2-G170A	***yCP β2-G170A: bortezomib***
**Crystal parameters**		
Space group	$P2_1$	$P2_1$
Cell constants	a= 134.8 Å	a= 134.2 Å
	b= 300.9 Å	b= 300.9 Å
	c= 144.5 Å	c= 144.1 Å
	β = 112.7 °	β = 112.3 °
CPs / AU[a]	1	1
**Data collection**		
Beam line	X06SA, SLS	X06SA, SLS
Wavelength (Å)	1.0	1.0
Resolution range (Å)[b]	50-2.8	50-2.8
	(2.9-2.8)	(2.9-2.8)
No. observations	684355	671723
No. unique reflections[c]	245599	249805
Completeness (%)[b]	94.4 (96.3)	96.5 (97.9)
R_{merge} (%)[b, d]	7.8 (47.5)	6.9 (45.3)
I/σ (I)[b]	11.1 (2.5)	10.6 (2.2)
**Refinement (REFMAC5)**		
Resolution range (Å)	15-2.8 Å	15-2.8 Å
No. refl. working set	233318	237314
No. refl. test set	12280	12491
No. non hydrogen	49867	49861
No. of ligand atoms	-	168
Solvent (H_2O, Mg^{2+}, Cl^-)	533	388
R_{work}/R_{free} (%)[e]	18.9 / 21.4	19.8 / 21.8
r.m.s.d. bond (Å) / (°)[f]	0.005 / 0.948	0.005 / 1.034
Average B-factor ($Å^2$)	61.6	70.0
Ramachandran Plot (%)[g]	97.8 / 1.9 / 0.3	97.7 / 2.0 / 0.3
PDB accession code	5BXL	5BXN

[a] Asymmetric unit
[b] The values in parentheses for resolution range, completeness, R_{merge} and I/σ (I) correspond to the highest resolution shell
[c] Data reduction was carried out with XDS and from a single crystal. Friedel pairs were treated as identical reflections
[d] $R_{merge}(I) = \Sigma_{hkl}\Sigma_j \mid I(hkl)_j - \langle I(hkl)\rangle \mid / \Sigma_{hkl} \Sigma_j I(hkl)_j$, where $I(hkl)_j$ is the jth measurement of the intensity of reflection hkl and <I(hkl)> is the average intensity
[e] $R = \Sigma_{hkl} \mid |F_{obs}| - |F_{calc}| \mid / \Sigma_{hkl} |F_{obs}|$, where R_{free} is calculated without a sigma cut off for a randomly chosen 5% of reflections, which were not used for structure refinement, and R_{work} is calculated for the remaining reflections
[f] Deviations from ideal bond lengths/angles
[g] Percentage of residues in favoured region / allowed region / outlier region

Table S2: Primers used in this study

Primer	*Sequence 5' → 3'*
Pup1_*Xba*I_for	CCA**TCTAGA**CAGTCTGCTTTGTAGTGGGG
Pup1_*Hin*dIII_rev	CCA**AAGCTT**ATATTACCCTGTTATCCCTAGC
G170A_for	GGAATGATTTGGGGTCT<u>GCT</u>TCTAATGTGGATGTGTG
G170A_rev	CACACATCCACATTAGA<u>AGC</u>AGACCCCAAATCATTCC
G170W_for	GGAATGATTTGGGGTCT<u>TGG</u>TCTAATGTGGATGTGTG
G170W_rev	CACACATCCACATTAGA<u>CCA</u>AGACCCCAAATCATTCC
5/6F	GAAACAGCTATGACCATGAT
5/6R	GACGGCCAGTGAATTGTAAT
Pup1_*Hin*dIII_for	CCA**AAGCTT**ATGGCAGGTTTATCGTTCGA
Pup1_ProA_*Sac*I_rev	CCA**GAGCTC**ATATTACCCTGTTATCCCTAGC
Mecl-1 Not1 fwd	ATTAGCGGCCGCGCCACCATGCTGAAGCAGGCAGTGG
Mecl-1 P2A rev	AGTTCGTGGCTCCGGAACCTTCCACCTCCATGGCCTGC
Mecl-1 P2A fwd	GCAGGCCATGGAGGTGGAAGGTTCCGGAGCCACGAACT
GFP EcoR1 rev	TAATGAATTCTTACTTGTACAGCTCGTCC

Triplets containing mutations are underlined and restriction sites are printed in bold.

Table S3: Plasmids used in this study

Plasmids	*Description*	*Source*
pRS315	CEN *LEU2*	Sikorski et al., 1989[1]
pRS425	*LEU2* 2μ	Christianson et al., 1992[2]
mP71	mP71	Gift from Wolfgang Uckert

Table S4: Yeast strains used in this study

Strains	*Relevant genotype*	*Source*
WCG4a	*MAT*α *leu2-3,112 ura3 his3-11,15 Can*^{S} *GAL2*	Heinemeyer et al., 1993[3]
YWH10	*pup1Δ::HIS3* [pRS316-*PUP1*]	Heinemeyer et al., 1997[4]
YUS4	*pup1-T1A*	Heinemeyer et al., 1997[4]
YEH38	*pup1Δ::HIS3* [pRS315-*PUP1-ProA-*H_7]	This study
YEH39	*pup1Δ::HIS3* [pRS315- *pup1-G170A-ProA-*H_7]	This study
YEH41	[pRS425- *pup1-G170A*]	This study
YEH42	[pRS425- *pup1-G170W*]	This study
YEH43	[pRS425-*PUP1-ProA-*H_7]	This study
YEH44	[pRS425- *pup1-G170A-ProA-*H_7]	This study
YEH45	[pRS425- *pup1-G170W-ProA-*H_7]	This study

All strains are isogenic with WCG4a.

Supplementary Methods

In situ test for chymotrypsin-like activity

Yeast strains, grown as patches on YPD plates, were replica plated onto sterile filter paper disks, lying on fresh YPD plates. The yeasts were grown on the filter papers for an additional 2 days at 30 °C. Cells were lysed by immersing the filter paper in 10 ml of chloroform for 15 min and subsequently covering the filter with 10 ml of overlay solution (1% (w/v) agar, 50 mM Tris-HCl pH 8.0, 300 µl of 10 mM Z-Gly-Gly-Leu-pNA (Carboxybenzyl-Gly-Gly-Leu-para-nitroanilide)). After incubation for 3 h at 30 °C, the following incubations were carried out: 10 ml of 0.1% (w/v) sodium nitrite solution in 1 M HCl for 5 min; 10 ml of 0.5% (w/v) ammonium sulfamate solution in 1 M HCl for 5 min; 10 ml of 0.05% (w/v) *N*-(1-naphthyl)ethylenediamine solution in 47% (v/v) ethanol for 10 - 30 min. Intense pink colouring is indicative of wt-like ChT-L activity towards the substrate Z-GGL-pNA.

Sucrose gradient density centrifugation

Sucrose gradient density centrifugation was performed as previously described[5]. Shortly, MEFs (stimulated for 24 h with 20 µg/ml recombinant mouse IFN-γ (Offenbach, Germany)) were lysed in 100 mM KCl containing 0.1% Triton-X-100 followed by sonication. The cleared lysate was loaded onto a gradient of 15–40% sucrose in 100 mM KCl buffer and centrifuged at 40,000 rpm for 16 h at 4°C in a Beckman Coulter (Krefeld, Germany) SW40Ti rotor. Afterwards, proteins in fractions of 600 µl were TCA-precipitated, separated by SDS-PAGE and MECL-1 was detected by Western blot.

Additional references

1. Sikorski, R.S. & Hieter, P. A system of shuttle vectors and yeast host strains designed for efficient manipulation of DNA in Saccharomyces cerevisiae. *Genetics* **122**, 19-27 (1989).
2. Christianson, T.W., Sikorski, R.S., Dante, M., Shero, J.H. & Hieter, P. Multifunctional yeast high-copy-number shuttle vectors. *Gene* **110**, 119-22 (1992).
3. Heinemeyer, W., Gruhler, A., Mohrle, V., Mahe, Y. & Wolf, D.H. PRE2, highly homologous to the human major histocompatibility complex-linked RING10 gene, codes for a yeast proteasome subunit necessary for chrymotryptic activity and degradation of ubiquitinated proteins. *J. Biol. Chem.* **268**, 5115-20 (1993).
4. Heinemeyer, W., Fischer, M., Krimmer, T., Stachon, U. & Wolf, D.H. The active sites of the eukaryotic 20 S proteasome and their involvement in subunit precursor processing. *J. Biol. Chem.* **272**, 25200-9 (1997).
5. Kremer, M., A. Henn, C. Kolb, M. Basler, J. Moebius, B. Guillaume, M. Leist, B. J. Van den Eynde and M. Groettrup (2010). "Reduced immunoproteasome formation and accumulation of immunoproteasomal precursors in the brains of lymphocytic choriomeningitis virus-infected mice." *J. Immunol.* **185**(9): 5549-5560.
